# Ferrocene‐Functionalized Black Phosphorus Nanoplatform Enables Targeted and Prolonged MRI Visualization of Atherosclerotic Plaques

**DOI:** 10.1002/advs.202503654

**Published:** 2025-06-05

**Authors:** Xiao Huang, Yuanyuan Zhao, Miao Zhou, Yukang Guo, Haiyun Wang, Yuanyuan Chen, Jian Peng

**Affiliations:** ^1^ Institute of Pharmaceutical Innovation Hubei Province Key Laboratory of Occupational Hazard Identification and Control School of Medicine Wuhan University of Science and Technology Wuhan 430065 China; ^2^ State Key Laboratory of Advanced Technology for Materials Synthesis and Processing Wuhan University of Technology Wuhan 430070 China; ^3^ Institute of Cardiovascular Diseases Hubei Province Key Laboratory of Occupational Hazard Identification and Control School of Medicine Wuhan University of Science and Technology Wuhan 430065 China

**Keywords:** atherosclerotic diagnosis, black phosphorus, contrast agents, magnetic resonance imaging, non‐metallic agents, stable radical

## Abstract

Despite the critical role of contrast‐enhanced magnetic resonance imaging (MRI) in clinical diagnostics, current contrast agents face dual challenges: metallic formulations provoke biosafety concerns while organic radicals exhibit transient imaging windows. To overcome these limitations, a radical‐engineered 2D black phosphorus (2D BP) nanoplatform is reported through atomic‐scale conjugation of aminoferrocene, creating a stable complex with enhanced electron spin resonance, named FcP. The covalent coupling strategy establishes persistent T_2_‐weighted contrast capability. Subsequent hyaluronic acid (HA) functionalization endows the HA‐FcP system with CD44‐specific targeting, achieving 1.7‐fold higher plaque accumulation than non‐targeted counterparts in apolipoprotein E‐deficient models. Remarkably, this nanostructure enables continuous MRI visualization of atherosclerotic lesions for 120 h post‐injection. Systematic biosafety evaluation demonstrates >90% cell viability and normal hematological parameters. This work pioneers a new paradigm for MRI contrast agents through spin state manipulation of 2D materials, providing a translational platform for precision cardiovascular diagnostics.

## Graphical abstract

Aminoferrocene is employed to modify black phosphorus, generating stable phosphorus radicals (FcP). The FcP is then functionalized with hyaluronic acid (HA) to produce the HA‐FcP radical complex, thereby developing a novel, highly efficient, sensitive, long‐lasting, and low‐heavy‐metal‐content radical contrast agent for targeted imaging of atherosclerotic plaques.

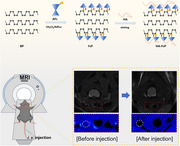

## 1 Introduction

In vivo imaging has become an indispensable tool in modern biomedical research and clinical practice, with magnetic resonance imaging (MRI) emerging as a cornerstone modality due to its non‐ionizing radiation, millimeter‐scale spatial resolution, and unparalleled soft tissue differentiation capabilities.^[^ ^1^ ^]^ Nevertheless, the intrinsically low sensitivity of proton relaxation‐based MRI necessitates the use of contrast agents to amplify signal differences between pathological and healthy tissues.^[^ ^2^ ^]^ Current clinical agents predominantly use paramagnetic gadolinium chelates (T_1_ agents) and superparamagnetic iron oxides (T_2_ agents), yet both categories face critical limitations. While gadolinium‐based contrast agents (GBCAs) account for over 40% of clinical MRI enhancements,^[^ ^3^ ^]^ their nephrotoxicity and the risk of gadolinium deposition in neural tissues have prompted FDA warnings and restricted usage in renal‐impaired patients.^[^ ^4^ ^]^ Although iron oxide nanoparticles present better biocompatibility, their tendency to induce hypersensitivity reactions and limited targetability restricts diagnostic precision.^[^ ^5^ ^]^ The pursuit of alternative metal‐free agents, such as nitroxide radicals, has been hampered by inherent instability and suboptimal relaxivity values.^[^ ^6^ ^]^ This impasse underscores the urgent demand for next‐generation contrast agents that synergize metal‐minimized composition with prolonged circulation stability and molecular targeting capability.

Atherosclerosis (AS), a chronic inflammatory disease characterized by arterial plaque formation, represents a critical diagnostic challenge.^[^ ^7^ ^]^ The vulnerability of these plaques, particularly those rich in macrophages, poses significant clinical risks.^[^ ^8^ ^]^ The CD44 receptor, overexpressed in activated plaque macrophages compared to normal endothelium, provides a molecular beacon for precise imaging.^[^ ^9^ ^]^ Hyaluronic acid (HA), a natural glycosaminoglycan with high CD44 affinity, has emerged as an ideal targeting moiety – its anionic surface not only enables specific ligand‐receptor recognition but also confers “stealth” properties through reduced opsonization.^[^ ^10^ ^]^ Recent advances in nanomaterial engineering have identified 2D black phosphorus (2D BP) as a superior theranostic platform, boasting exceptional surface area, tunable electronic properties, and excellent biocompatibility.^[^ ^11^ ^]^

**Scheme 1:**
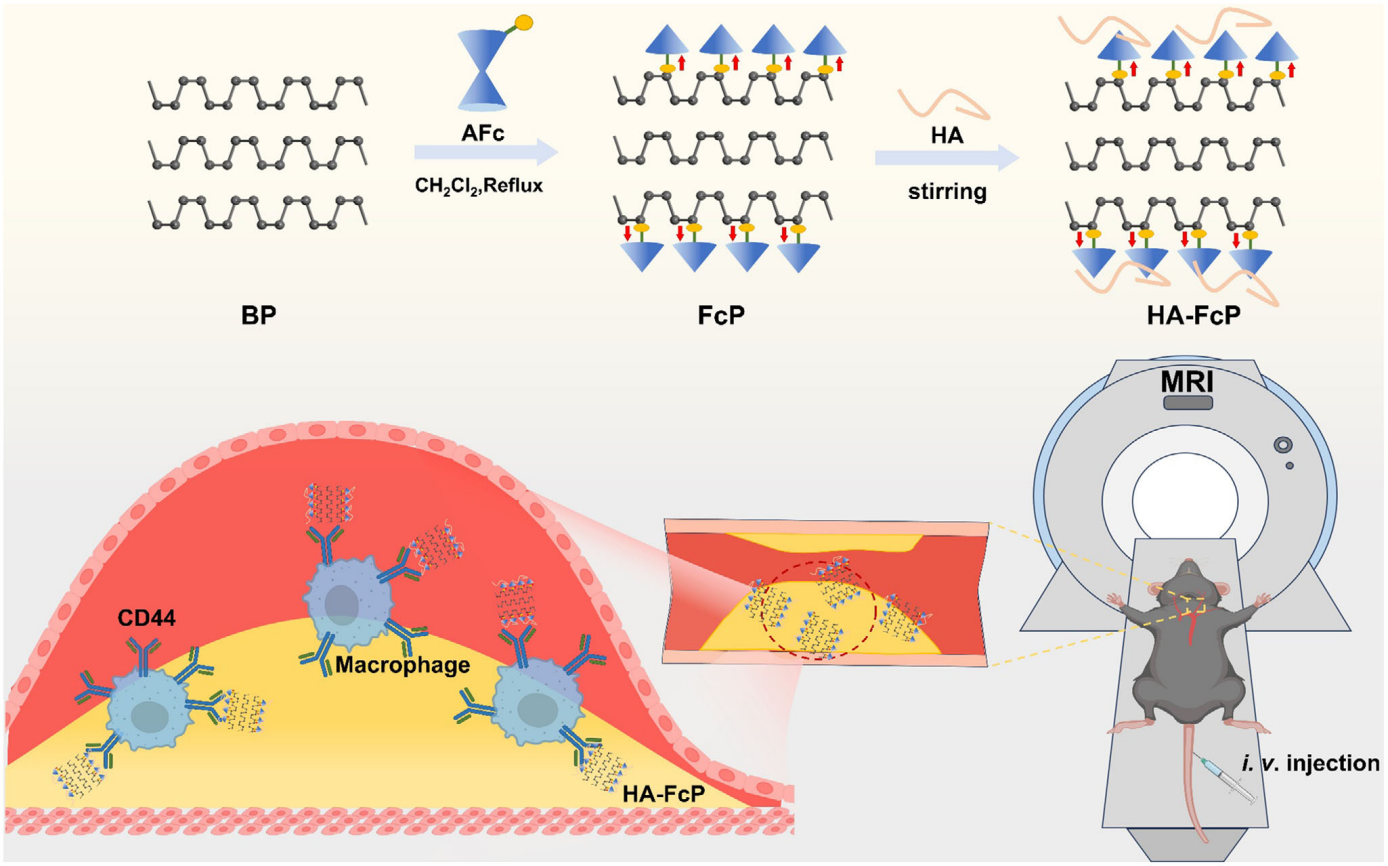
Schematic illustration of HA‐FcP application for targeted MRI detection of atherosclerotic plaques.

Building upon previous work demonstrating BP's therapeutic potential in AS,^[^ ^12^ ^]^ we developed a novel contrast agent through surface modification of BP with aminoferrocene (AFc) to create stable radicals, named FcP, followed by HA functionalization for targeted plaque imaging (**Scheme** **1**). This HA‐modified complex demonstrates (HA‐FcP) selective targeting of CD44‐positive cells within atherosclerotic plaques, superior imaging performance compared to BP, and sustained MRI signal detection for up to 120 h. Comprehensive pharmacological evaluation revealed no significant alterations in liver/kidney function or inflammatory markers. This metal‐minimized design paradigm establishes a new framework for developing targeted, durable, and biosafe contrast agents in molecular MRI.

## 2 Results and Discussion

### 2.1 Preparation and Characterization of FcP

**Figure 1:**
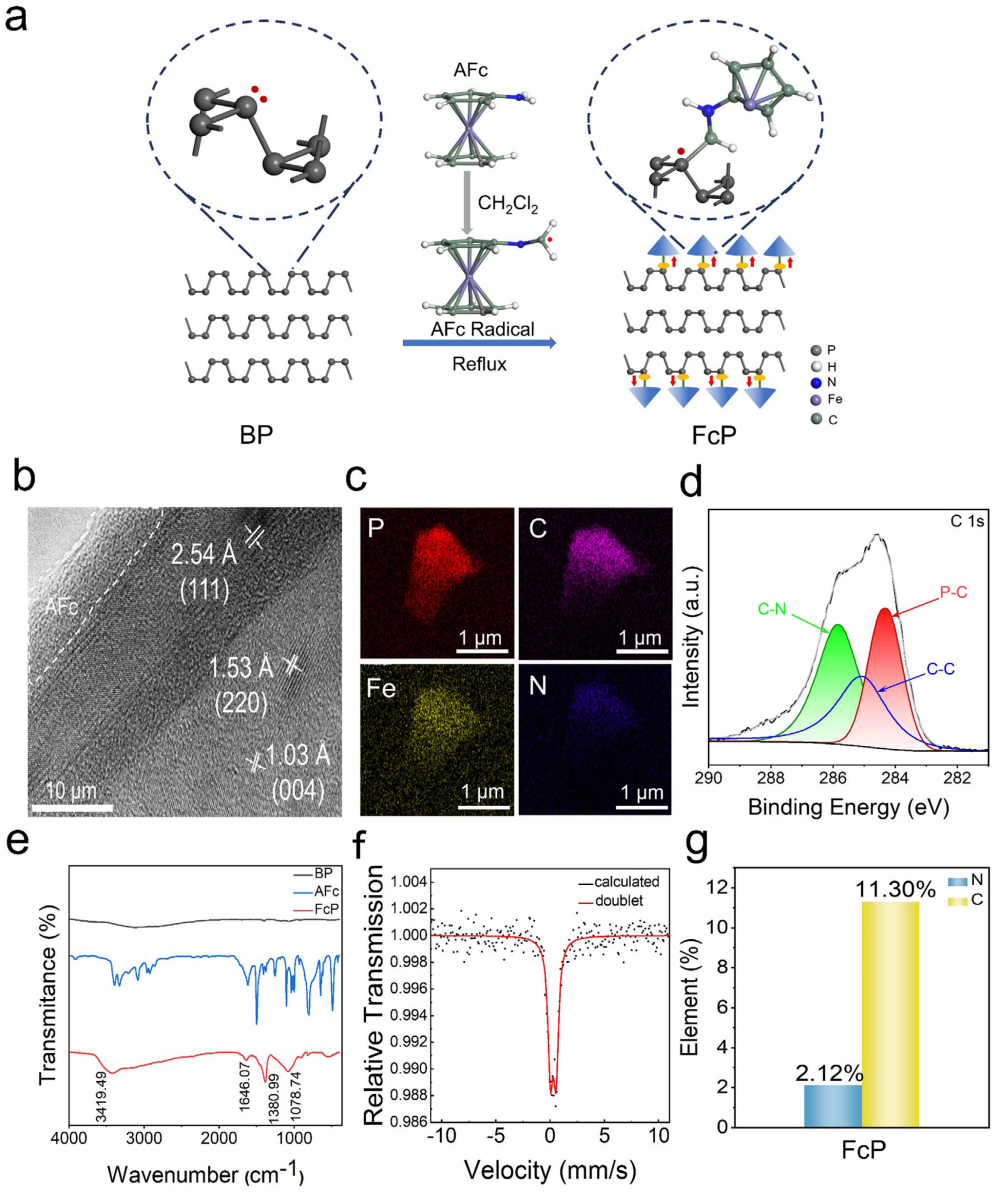
Structural and chemical characterization of FcP. a) Schematic illustration of the synthetic route for FcP production from layered BP. b) HRTEM image of FcP. c) Energy‐dispersive X‐ray spectroscopy (EDS) elemental mapping of a single FcP nanosheet. d) X‐ray photoelectron spectroscopy (XPS) analysis of C 1s core‐level spectra in FcP. e) Comparative Fourier‐transform infrared (FTIR) spectra of BP, AFc, and FcP. f) Mössbauer spectroscopy analysis of FcP. g) Quantitative analysis of carbon and nitrogen content (wt.%) in FcP.

In this work, exfoliated BP was synthesized via liquid‐phase exfoliation of bulk BP in dimethylformamide (DMF). The FcP complex was subsequently prepared through reaction of 40 mL BP (1 mg mL^−1^) with AFc in dichloromethane at 100 °C for 10 h (**Figure** **1**a). Post‐modification, FcP demonstrated enhanced ambient stability (Figure S1, Supporting Information), with maintained hydrodynamic size. Scanning electron microscopy (SEM) imaging (Figure S2, Supporting Information) revealed characteristic 2D lamellar structures with dimensions of 2 – 5 µm. High‐resolution transmission electron microscopy (HRTEM) analysis (Figure S3, Supporting Information) confirmed the crystalline integrity of individual nanosheets, while atomic‐resolution TEM (Figure 1b) identified distinct lattice spacings of 2.54 Å, 1.53 Å and 1.03 Å, corresponding to the (111), (220) and (004) crystallographic planes of BP, respectively. Amorphous domains (white dashed circles) were ascribed to AFc coordination.

X‐ray diffraction (XRD) patterns (Figure S4, Supporting Information) of both BP and FcP matched the orthorhombic phase (JCPDS No. 73–1358), confirming structural preservation during functionalization. Energy‐dispersive X‐ray spectroscopy (EDS) elemental mapping (Figure 1c) revealed homogeneous spatial distributions of P, C, N, and Fe, verifying successful AFc conjugation. Atomic force microscopy (AFM) measurements (Figure S5, Supporting Information) yielded an average nanosheet thickness of 18–24 nm. Dynamic light scattering (DLS) analysis showed FcP's hydrodynamic diameter (1624.91 nm) slightly exceeded that of pristine BP (Figure S6a,b, Supporting Information). Zeta potential measurements demonstrated charge modulation from −13.76 mV (BP) to −7.62 mV (FcP), consistent with partial neutralization of BP's negative surface charge by protonated amino groups in AFc (Figure S6c, Supporting Information). To investigate the amount of AFc modification on BP, we conducted thermogravimetric analysis (TGA). TGA quantified AFc loading as 12.17 wt.%, based on organic decomposition between 150–460 °C (Figure S7, Supporting Information). Collectively, these multimodal characterization results confirm the successful construction of AFc‐functionalized BP nanosheets with retained crystallinity and enhanced stability.

### 2.2 Covalent Functionalization of BP via P‐C Bond Formation with Aminoferrocene

To investigate the surface chemical bonding information, X‐ray photoelectron spectroscopy (XPS) analysis of both pristine and AFc‐functionalized BP (FcP) revealed significant chemical modifications. The survey spectrum of FcP showed substantial Fe incorporation (Figure S8a,c, Supporting Information), confirming successful covalent modification. High‐resolution C 1s and P 2p spectra exhibited characteristic bonding signatures at ≈284 eV (C‐P) and ≈133 eV (P‐C), respectively (Figures 1d and S8b, Supporting Information), consistent with established covalent functionalization mechanisms.^[^ ^13^ ^]^ The absence of these features in pristine BP (Figure S8d, Supporting Information) and the emergence of new vibrational modes in Fourier‐transform infrared (FTIR) spectroscopy provided complementary evidence for covalent bonding (Figure 1e). The FTIR spectrum of FcP displayed distinct absorption bands at 3419 cm^−1^ (N‐H stretching), 1646 cm^−1^ (C═C vibration), 1381 cm^−1^ (C‐N bending), and 1079 cm^−1^ (C‐H bending), corresponding to AFc modification.^[^ ^14^ ^]^ Ultraviolet‐visible (UV–Vis) spectroscopy demonstrated modified optical properties in FcP, with a red shift observed in the characteristic peak of ferrocene at 440 nm (Figure S9, Supporting Information).^[^ ^15^ ^]^ Raman analysis revealed systematic red shifts of 5 cm^−1^ (A^1^_g_ mode) and 7 cm^−1^ (A^2^_g_ and B_2g_ modes), indicating chemical‐induced lattice strain and suppressed P‐atom oscillations (Figure S10, Supporting Information).

We further investigated the valence state changes of iron after the covalent binding of AFc with BP. Mössbauer spectroscopy of FcP exhibited an asymmetric doublet (Isomer Shift (IS) = 0.29 mm s^−1^) characteristic of ferrocene derivatives (Figure 1f, Table S1, Supporting Information). Elemental analysis quantified the surface composition as 11.30% C and 2.12% N (C/N ≈ 6, Figure 1d), while Fe 2p XPS showed peaks at 712.05 eV (2p_3/2_) and 725.69 eV (2p_1/2_), confirming the species of oxidized Fe^3+^ (Figure S8a, Supporting Information).^[^ ^16^ ^]^ This electronic reorganization from 3d^6^ (Fe^2+^) to 3d^5^ (Fe^3+^) configuration suggests structural rearrangement during AFc attachment via P‐C‐N bonding. The oxidation mechanism involves P radical formation‐induced electron withdrawal from the metallocene ring, leading to symmetry breaking and subsequent ring dissociation.^[^ ^17^ ^]^ Brunauer‐Emmett‐Teller (BET) analysis revealed a surface area of 12.05 m^2^ g^−1^ (Figure S11, Supporting Information), corresponding to 6.28 AFc molecules nm^−2^. The experimental P:AFc molar ratio of 4.38:1 closely matched the theoretical 4:1 ratio for monolayer coverage (Figures S12,S13, Supporting Information), confirming comprehensive surface functionalization.

### 2.3 Characterisation of the Properties of FcP Radicals

**Figure 2:**
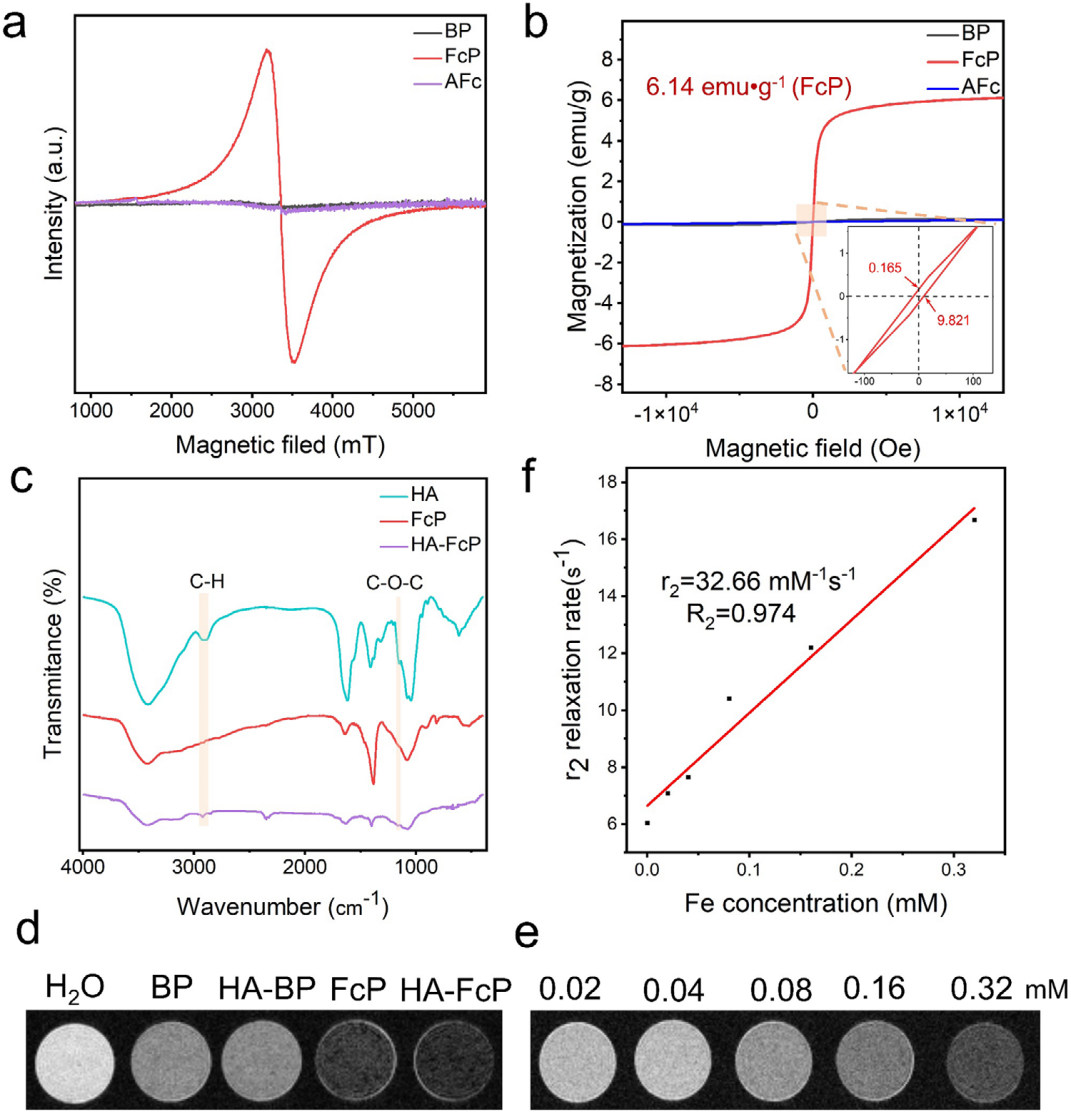
Characterization and magnetic properties of the FcP radicals. a) EPR spectra of BP, FcP, and AFc. b) Vibrating sample magnetometry (VSM) of FcP, AFc, and BP. c) FTIR spectra of HA, FcP, and HA‐FcP. d) T_2_‐weighted images of H_2_O, BP, HA‐BP, FcP, and HA‐FcP. e) T_2_ images of different Fe concentrations of HA‐FcP. (f) Linear variation curves of r_2_ relaxation rate with concentration for different concentrations of HA‐FcP.

To investigate the radical generation in FcP nanocomposites, we conducted electron paramagnetic resonance (EPR) spectroscopy analysis of AFc, BP, and FcP. Notably, a characteristic EPR signal emerged exclusively in FcP (*g* = 2.003, **Figures** **2**a and S14, Supporting Information), confirming the successful formation of phosphorus‐centered radicals.^[^ ^18^ ^]^ Intriguingly, bare BP and AFc remained EPR‐silent under identical conditions, highlighting the effect of covalent modification in radical generation. Magnetic susceptibility measurements revealed unconventional room‐temperature ferromagnetism in FcP (Figure 2b). Quantitative analysis through Brillouin function fitting yielded a saturation magnetization (Ms) of 6.14 emu g^−1^. Complementary inductively coupled plasma mass spectrometry (ICP‐MS) data quantified the iron content at 2.44 wt.%, equivalent to an impressive Fe‐specific Ms of up to 251.64 emu g^−1^. Despite the much higher total iron content (72.4 wt.%) and MS (70 emu g^−1^) of conventional contrast agents SPIONs, the Ms per unit mass of iron in FcP is still 2.6 times to that of SPIONs (96.69 emu g^−1^).^[^ ^19^ ^]^ This magnetic enhancement stems from electronic restructuring induced by covalent BP modification. While AFc adheres to the 18‐electron configuration typical of ferrocene derivatives (Fe^2+^: 3d^6^, paired electrons),^[^ ^20^ ^]^ the hybridization‐driven electronic reorganization to Fe^3+^ (3d^5^) generates unpaired spins capable of ferromagnetic alignment. Furthermore, 2D superexchange interactions mediated by P radicals within the BP plane synergistically amplify the magnetic response.^[^ ^14^ ^]^ Collectively, these advancements culminate in an MRI contrast agent featuring exceptional magnetic performance coupled with drastically reduced heavy metal content.

To optimize biological performance, we designed HA‐FcP by modifying the surface with HA. TEM visualized nanoscale vesicular structures decorating the HA‐FcP surface (Figure S15, Supporting Information). FTIR spectroscopy verified successful functionalization via characteristic C─O─C stretching vibrations (1045 cm^−1^, Figure 2c). The hydrodynamic diameter of HA‐FcP showed no significant changes over a 14‐day period, indicating excellent stability of HA‐FcP (Figure S16a, Supporting Information). UV–Vis spectroscopy, XRD, and Raman spectroscopy all demonstrated that the original structure and properties of FcP remained unaltered after modification with HA (Figure S16b–d, Supporting Information). Comparative MRI studies at equivalent concentrations (2 mg mL^−1^) demonstrated HA‐FcP's superior T_2_‐weighted contrast relative to BP and HA‐BP controls (Figure 2d). Concentration‐dependent imaging revealed progressive signal darkening with increasing HA‐FcP concentration (Figure 2e). Quantitative analysis yielded a transverse relaxivity (r_2_) of 32.66 mM^−1^ s^−1^ for HA‐FcP (Figure 2f). The transverse relaxivity of HA‐FcP exceeds that of certain iron‐based contrast agents reported in the current literature.^[^ ^21^ ^]^ Moreover, under equivalent iron concentrations, HA‐FcP exhibits superior MRI contrast enhancement compared to the typical iron‐based MRI contrast agent SPIONs, suggesting its potential as a next‐generation MRI probe with enhanced sensitivity (Figure S17, Supporting Information).

### 2.4 Screening AS Plaques Using HA‐FcP Mediated MRI Too

**Figure 3:**
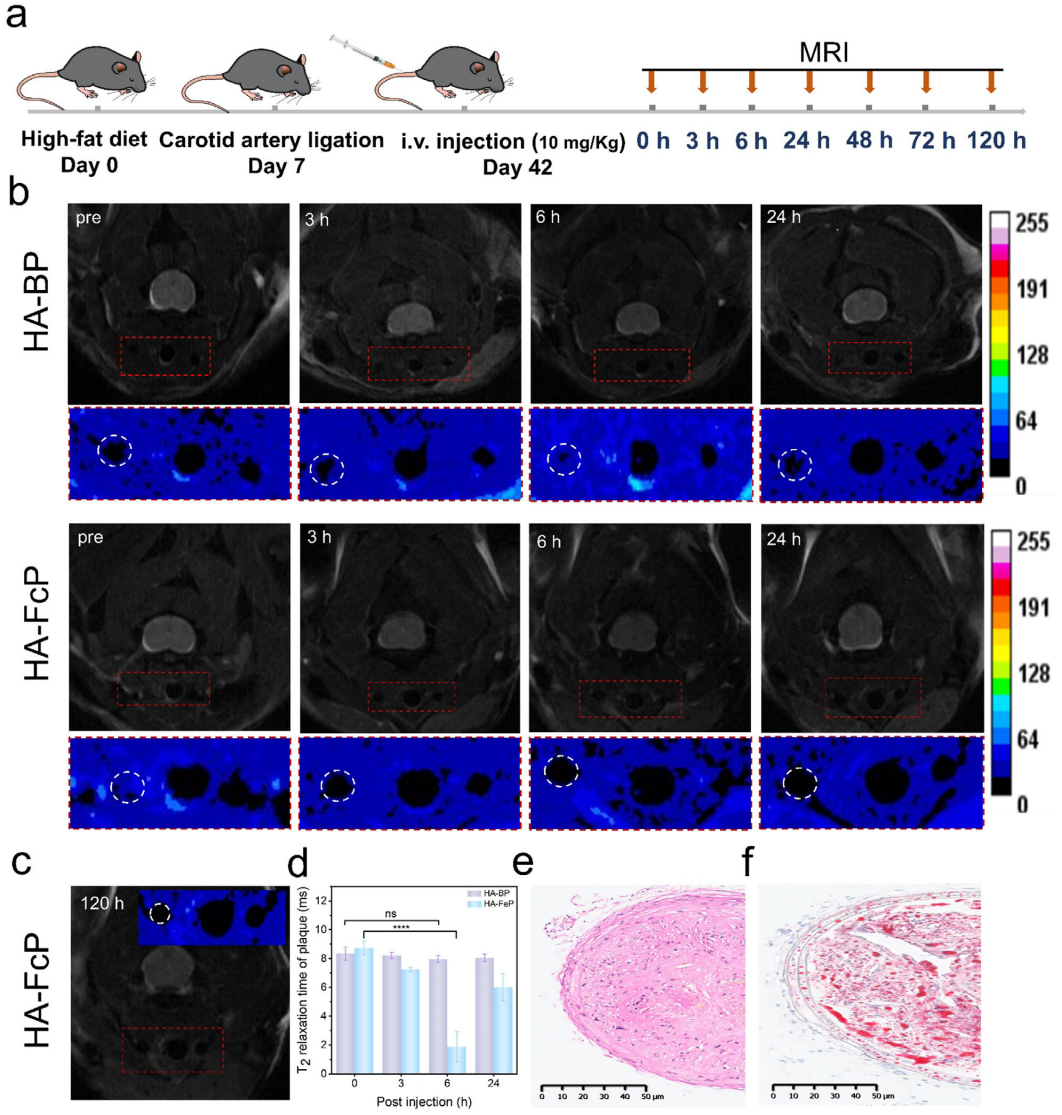
Accumulation and imaging of HA‐FcP in moderate aortic AS models. a) Schematic illustration of in vivo MRI detection of carotid artery plaques. b) MRI images of carotid artery plaques in mice at different time points. The vessel wall is marked with a white circle, and the plaque is displayed within the white circle. c) MRI of the carotid aorta in AS at 120 h after injection of HA‐FcP. d) Quantification of T_2_ relaxation times in the plaques (n = 5). e) Hematoxylin and eosin (H&E) staining of plaques after MRI detection. f) Oil Red O staining of plaques after MRI detection. Data are presented as mean ± standard deviation (SD). Statistical differences were analyzed by Student's *t*‐test: ^****^ p< 0.0001, ns: not significant.

MRI, renowned for its submillimeter spatial resolution and exceptional soft‐tissue penetration depth, has emerged as a cornerstone noninvasive imaging modality for atherosclerotic plaque characterization.^[^ ^22^ ^]^ To evaluate the performance of contrast agent, we established an AS model in apolipoprotein E‐deficient (ApoE^‐/‐^) mice through partial ligation of the left carotid artery (LCA) coupled with a 5‐week high‐fat diet, while maintaining the right carotid artery (RCA) as untreated controls (Figure S18, Supporting Information), all animal experiments were approved by the Wuhan University of Science and Technology (ethicalapproval number 2023038). Tail vein injection of hyaluronic acid‐modified black phosphorus (HA‐BP) or HA‐FcP enabled MRI assessment at predetermined intervals (3, 6, 24, 48, 72, and 120 h post‐injection; **Figure** **3**a).

Notably, HA‐FcP demonstrated superior contrast‐enhanced imaging capabilities with sustained signal enhancement in atherosclerotic lesions. Quantitative analysis revealed no significant T_2_‐weighted signal alterations in HA‐BP‐treated plaques throughout the observation period, confirming its inherent MRI‐inert properties (Figure 3b). In stark contrast, HA‐FcP administration induced pronounced signal amplification within 6 h, with persistent contrast retention maintaining signal intensity at 120 h (Figures 3c,d, and S19, Supporting Information). Following MRI, mice were sacrificed, and histological examination results of the carotid arteries were consistent with the pathological and ultrasonic evaluation results (Figures 3e,f, and S20, Supporting Information). Moreover, Prussian blue staining performed on carotid artery sections obtained 120 h after injection demonstrated the continued presence of iron ion accumulation within the plaque regions, thereby furnishing additional confirmation that the MRI signal remained discernible at the 120‐h mark (Figure S21, Supporting Information). This iron‐engineered nanoformulation achieves dual optimization through: (i) Hyaluronan‐mediated active targeting of CD44‐overexpressing activated macrophages; (ii) Ferromagnetic enhancement enabling prolonged imaging windows. The demonstrated 120‐h visualization capacity positions HA‐FcP as a transformative platform for plaque monitoring and therapeutic evaluation in atherogenesis.

### 2.5 Plaque Targeting and Biodistribution of HA‐FcP

**Figure 4:**
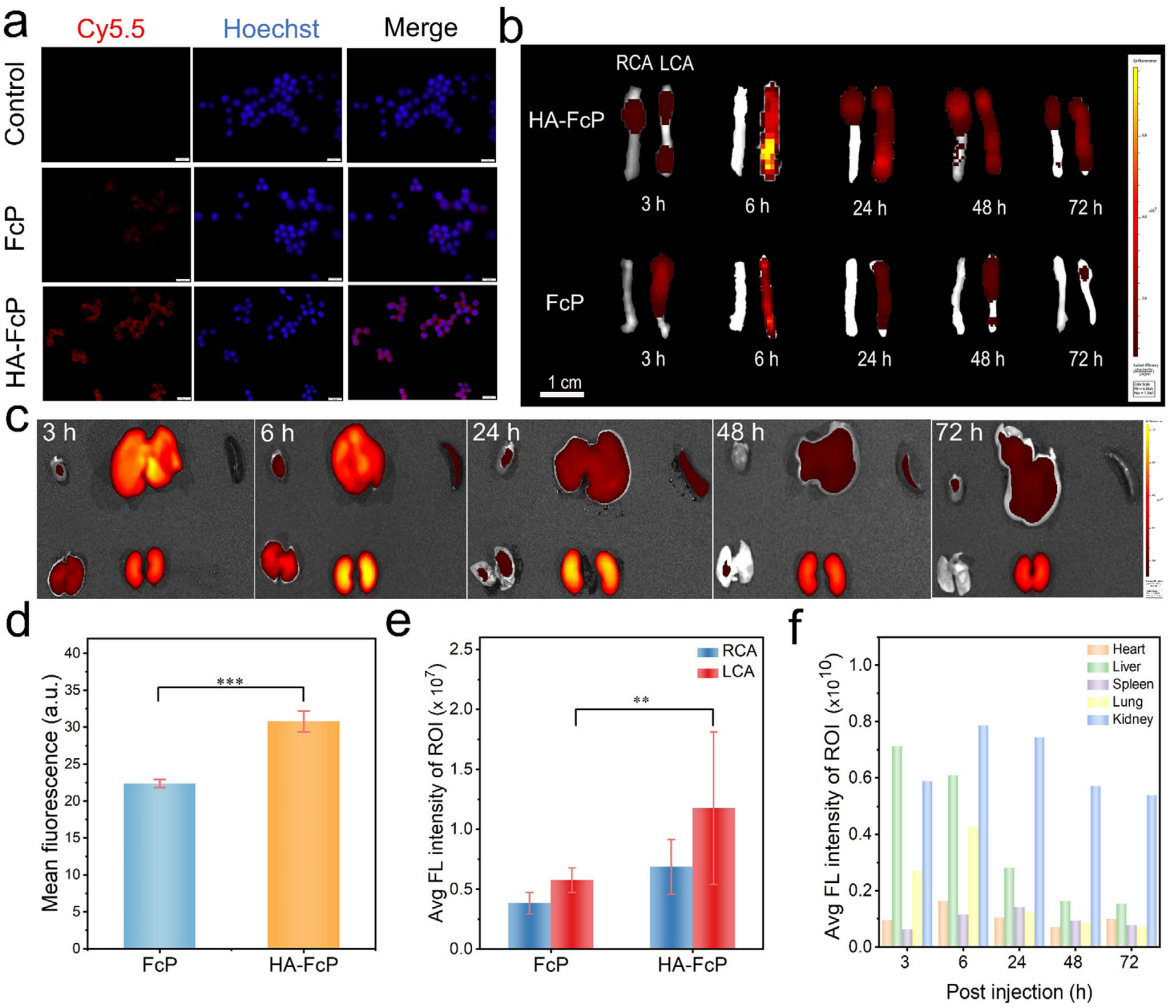
Plaque targeting and biodistribution of HA‐FcP. a) Representative fluorescence images of activated macrophages cultured with different materials. Scale bar: 20 µm. b) Ex vivo fluorescence images of RCA and LCA. c) Ex vivo fluorescence image of the resected major organ. d) Quantitative analysis of the fluorescent intensity in (a). e) Quantitative analysis of the fluorescent intensity in (b) at 6 h (n = 3). f) Quantitative analysis of fluorescence intensity in (c). Data are presented as mean ± SD. Statistical differences were analyzed by Student's *t*‐test: ^**^p < 0.01, ^***^p < 0.001 were considered highly significant.

Activated macrophages serve as critical proinflammatory mediators in AS plaque development, driving both lesion initiation and progression.^[^ ^23^ ^]^ The molecular recognition between HA and CD44 receptors overexpressed on these activated macrophages has been strategically exploited for targeted theranostics in AS management.^[^ ^24^ ^]^ To validate this targeting mechanism, we first established an in vitro model using lipopolysaccharide (LPS)‐induced proinflammatory macrophages. Sulfo‐Cyanine5.5 (Cy5.5)‐labeled FcP and HA‐FcP complexes were then incubated with macrophages, revealing significantly enhanced cellular uptake of HA‐FcP‐Cy5.5 compared to its unmodified counterpart through quantitative fluorescence imaging (**Figure** **4**a,d).

This targeting specificity was further corroborated by in vivo imaging system (IVIS) tracking following intravenous administration. Time‐dependent biodistribution analysis demonstrated that 6 h after injection, HA‐FcP gradually accumulated at the AS plaques (Figure S22, Supporting Information), with LCA fluorescence intensity in AS‐model mice surpassing both the contralateral RCA. Ex vivo IVIS quantitative analysis of excised blood vessels showed that the fluorescence signal of HA‐FcP‐Cy5.5 increased over time, reaching a peak at 6 h post‐injection, with an accumulation at the LCA plaque site that was 1.7‐fold higher than that of FcP‐Cy5.5 (Figure 4b,e). Notably, the HA‐modified nanoparticles exhibited characteristic biodistribution patterns of nanomedicines, showing transient hepatic and renal accumulation followed by systemic clearance (Figure 4c,f). This dual‐validation through imaging modalities confirms HA‐FcP's macrophage‐targeting precision across cellular and organismal levels, establishing a robust platform for molecular MRI detection of AS plaques. The time‐resolved targeting profile further informs optimal imaging window selection for clinical translation.

### 2.6 Biosafety Evaluation of HA‐FcP

**Figure 5:**
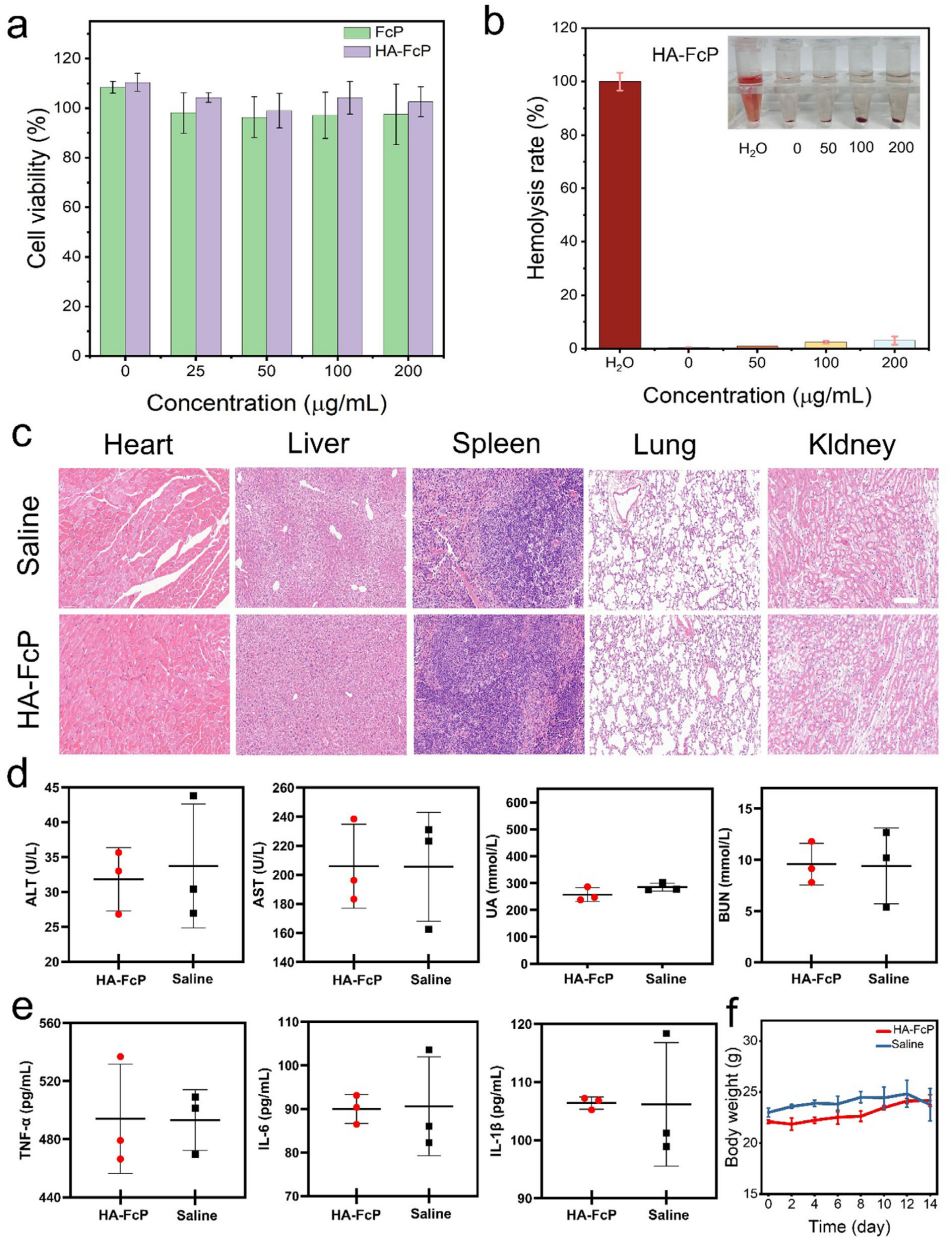
Biosafety evaluation of HA‐FcP. a) Viability of macrophages after incubation with FcP, HA‐FcP at different concentrations for 24 h. b) Hemolysis test of HA‐FcP at different concentrations. Inset: Solution images at different concentrations. c) H&E staining of major organs in C57BL/6 mice. Scale bar: 100 µm. d) Liver function biomarkers (ALT and AST) and kidney function biomarkers (BUN and UA) in the serum of C57BL/6 mice. e) Levels of inflammatory cytokines (TNF‐α, IL‐6, and IL‐1β) in the serum of C57BL/6 mice. f) Changes in the body weight of C57BL/6 mice. Data are presented as mean ± SD (n = 3).

The biosafety of nanomaterials is a crucial prerequisite for their practical application in AS diagnosis. Therefore, the cytotoxicity of HA‐FcP was investigated. As shown in **Figure** **5**a, the results of the 3‐(4,5‐dimethylthiazol‐2‐yl)‐2,5‐diphenyltetrazolium bromide) (MTT) assay indicates that HA‐FcP at a concentration of 200 µg mL^−1^ did not significantly reduce macrophage viability. Furthermore, HA modification effectively mitigated the cytotoxicity of FcP, demonstrating that HA‐FcP exhibits excellent cytocompatibility.

We further evaluated the blood compatibility of HA‐FcP. The hemolysis rate remained below 5% at concentrations up to 200 µg mL^−1^, indicating that HA‐FcP possesses good hemocompatibility in the bloodstream (Figure 5b). For a comprehensive evaluation of the in vivo biocompatibility of HA‐FcP, two groups of C57BL/6 mice were administered saline or HA‐FcP (10 mg kg^−1^) via tail vein injection. Histological examination of the heart, liver, spleen, lungs, and kidneys revealed no significant tissue damage in HA‐FcP‐treated mice (Figure 5c). The routine blood tests conducted on mice showed no significant differences in white blood cell count (WBC), red blood cell count (RBC), platelet count (PLT), and hemoglobin concentration (HGB) compared with the normal saline group, indicating that HA‐FcP has good blood compatibility (Figure S23, Supporting Information). Additionally, serum biomarkers of liver function (alanine aminotransferase (ALT), and aspartate aminotransferase (AST)), renal function (blood urea nitrogen (BUN), serum creatinine (SCR)), and inflammatory cytokine levels (tumor necrosis factor‐α (TNF‐α), interleukin‐6 (IL‐6), and interleukin‐1β (IL‐1β)) in HA‐FcP‐treated mice were comparable to those in the saline‐treated group (Figures 5d,e and Figure S24, Supporting Information). Moreover, body weight changes in HA‐FcP‐treated mice were similar to those in the saline group over a 15‐day observation period (Figure 5f).

## 3 Conclusion

In summary, we have successfully engineered a HA‐FcP nanoplatform that enables targeted, persistent, and biologically safe magnetic resonance imaging of atherosclerotic plaques. The design capitalizes on the specific binding affinity between HA ligands and overexpressed CD44 receptors on plaque‐associated macrophages, achieving remarkable targeting efficiency. Systematic biosafety evaluations confirm the excellent biocompatibility of HA‐FcP, with hematological parameters and histological analyses revealing negligible systemic toxicity. Notably, the engineered system demonstrates superior contrast performance in vivo, maintaining diagnostically relevant T_2_‐weighted signal attenuation for up to 120 h post‐administration. This prolonged retention time, combined with the intrinsic high magnetic susceptibility of FcP, positions HA‐FcP as a dual‐functional theranostic agent. Our findings not only establish HA‐FcP as a durable MRI contrast candidate but also illuminate its potential to utilize its superior magnetic properties for magnetothermal therapy in treating AS plaques. This nanoplatform design strategy opens new avenues for precise diagnosis and treatment of cardiovascular diseases.

## 4 Experimental Section

### Materials

Red phosphorus (RP), tin and tin iodide (SnI_4_), aminoferrocene (AFc), hyaluronic acid (HA), Lipopolysaccharide (LPS), sulfo‐Cyanine5.5 (Cy5.5) were purchased from Macklin Biochemical Co. Ltd (Shanghai, China). Dimethylformamide (DMF), dichloromethane (CH_2_Cl_2_), and other reaction reagents were purchased from Sinopharm Chemical Reagent Co., Ltd. (Shanghai, China). All chemicals were used without further purification. 3‐(4,5‐dimethylthiazol‐2‐yl)‐2,5‐diphenyltetrazolium bromide (MTT), Hoechst 33 342, and Dimethyl sulfoxide (DMSO) were obtained by the biosharp Co., Ltd (Guangzhou, China).

### Preparation of FcP

Bulk black phosphorus was prepared by a facile low‐pressure transport method. Subsequently, 2D layered black phosphorus was prepared via liquid‐phase exfoliation. Under a nitrogen atmosphere, a black phosphorus suspension dissolved in N,N‐dimethylformamide (DMF) was stirred with dichloromethane (CH_2_Cl_2_) at room temperature. Subsequently, aminoferrocene (AFc) was added to the above mixture, which was then heated to 100 °C with continuous stirring. The final product was collected by centrifugation and washed with ethanol to obtain FcP.

### Preparation of HA‐FcP

Mix the HA solution with the aqueous solution of FcP by stirring. After stirring overnight, centrifugation and washing were performed to obtain the HA‐FcP sample.

### Characterization of Samples

The ultraviolet‐visible (UV–Vis) absorption spectra of the samples were measured using a Shimadzu UV‐1900i spectrometer. Raman spectroscopic data were collected with an Xplora PLUS spectra, infrared spectroscopic analysis was performed using an INVENIOR Fourier transform infrared (FTIR) spectrometer. The crystal structures of the samples were characterized by a Rigaku/SmartLab SE X‐ray diffractometer (XRD). Microscopic images of the samples were obtained using a ThermoFisher/Apreo S HiVac scanning electron microscope (SEM) at an accelerating voltage of 10 kV. The composition of FcP was analyzed using an AXIS SUPRA+ X‐ray photoelectron spectrometer (XPS) equipped with a Mg Kα X‐ray source. Thermogravimetric analysis (TGA) was conducted under an argon atmosphere at a heating rate of 10 K min^−1^ using a 449C/449F3 thermogravimetric analyzer. Dynamic light scattering (DLS) and Zeta potential measurements were performed using a Malvern Nano−ZS instrument. Atomic force microscopy (AFM) images were acquired by scanning in contact mode in air using a Bruker/Dimension ICON. Electron paramagnetic resonance (EPR) spectra were measured with a Bruker EMXplus‐6/1 spectrometer, and magnetic data were collected at room temperature using a Lake Shore/8604 physical property measurement system.

### Cytotoxicity Evaluation

Macrophage viability was assessed using the MTT assay following 24‐h exposure to varying concentrations (0‐200 µg mL^−1^) of HA‐FcP and FcP in 96‐well culture plates. Cells were seeded at a density of 1 × 10^4^ cells well^−1^ and maintained under standard culture conditions (37 °C, 5% CO_2_) prior to treatment.

### Cellular Uptake Analysis

LPS‐activated RAW 264.7 macrophages were plated in 24‐well chamber slides and treated with either Cy5.5‐HA‐FcP or Cy5.5‐FcP for 4 h at 37 °C. Following PBS washing, cells were fixed with 4% paraformaldehyde, counterstained with Hoechst for nuclear visualization, and subjected to confocal laser scanning microscopy (CLSM; Zeiss LSM 880) analysis. Fluorescence intensity quantification was performed using ImageJ software.

### Animal Model

All animal procedures were authorized by the Wuhan University of Science and Technology (Ethical Approval No. 2023038). Male ApoE‐deficient (ApoE^‐/‐^) mice aged 6–8 weeks were procured from Huachuang Sino Biotechnology Co., Ltd. (Jiangsu, China). Animals were maintained in individually ventilated cages under specific pathogen‐free conditions with a 12‐h light/dark cycle and fed a high‐fat diet to induce hyperlipidemia. On day 14 of the protocol, left common carotid artery bifurcation was surgically ligated to initiate atherosclerotic plaque formation, while the right left common carotid artery underwent a sham operation as control.

### In Vivo Localization of HA‐FcP

Fourteen days following partial carotid ligation surgery, mice were randomly allocated into two cohorts and administered intravenous injections of either Cy5.5‐FcP or Cy5.5‐HA‐FcP at a dose of 10 mg kg^−1^ body weight. At designated time intervals, the left and right carotid arteries were harvested and subjected to in vivo fluorescence imaging using a PerkinElmer IVIS Spectrum system. The accumulation of HA‐FcP in atherosclerotic lesions was quantitatively assessed based on the fluorescence intensity signals detected by the imaging system. Quantitative analysis of nanoparticle accumulation was performed by measuring region‐of‐interest (ROI) fluorescence intensity using Living Image software.

### In Vivo MRI Screening of Plaques in ApoE^‐/‐^ Mice

Images were obtained using a 9.4 T (Bruker 94/30 USR) small animal magnet with a birdcage coil having a diameter of 30 cm. Mice were anesthetized, and respiration was monitored (MP150, Biopac, Goleta, CA). Intravenous injection of 10 mg kg^−1^ HA‐BP and HA‐FcP via the tail vein for in vivo MRI.

### Histopathological Examination

After completing distinct intervention protocols, experimental mice were humanely euthanized on day 42, and the left common carotid arteries were promptly isolated. The excised vascular tissues were fixed and subsequently subjected to hematoxylin‐eosin (H&E) staining to evaluate histopathological features of atherosclerotic plaques, along with Oil Red O staining for quantitative assessment of lipid accumulation within the plaques. Stained sections were then imaged using an optical microscope for morphological analysis.

### Hemolysis Assay

Suspensions of HA‐FcP in phosphate‐buffered saline (PBS) were mixed with murine red blood cells at varying concentration gradients, using deionized water as the positive control. Following 1‐h incubation at 37 °C, the mixtures were centrifuged to separate the components. The absorbance of the resulting supernatant was then measured at 576 nm using a microplate reader.

### Biodistribution Analysis

2 groups of 6‐week‐old female C57BL/6 mice (n = 3 in each group) were *i.v*. injected with saline and HA‐FcP, respectively. Body weight was continuously monitored for 15 days following drug administration. Upon experimental termination, major organs were harvested from each group and subjected to H&E staining for histopathological evaluation. Additionally, peripheral blood was collected for serum separation, and biochemical assays were performed using an automated analyzer to assess hepatic function markers, renal function markers, and inflammatory cytokine levels.

### Statistical Analysis

All data were expressed as “mean ± standard deviation (SD)”. Data analysis by Student's t‐test: ^*^p < 0.05, ^**^p < 0.01, ^***^p < 0.001, ^****^p < 0.0001, ns: not significant.

## Conflict of Interest

The authors declare no conflict of interest.

## Supporting information



Supporting Information

## Data Availability

The data that support the findings of this study are available on request from the corresponding author. The data are not publicly available due to privacy or ethical restrictions.;
