# Supplementary material for: Ferrocene‐Functionalized Black Phosphorus Nanoplatform Enables Targeted and Prolonged MRI Visualization of Atherosclerotic Plaques
Source: Adv Sci (Weinh). 2025 Jun 5;12(32):e03654. doi: 10.1002/advs.202503654 (PMC12407352; doi:10.1002/advs.202503654)
Supplement: Supplementary file 1 — Supporting Information [file ADVS-12-e03654-s001.pdf]

ADVANCED SCIENCE
Open Access

Supporting Information

for *Adv. Sci.*, DOI 10.1002/advs.202503654

Ferrocene-Functionalized Black Phosphorus Nanoplatform Enables Targeted and Prolonged MRI Visualization of Atherosclerotic Plaques

*Xiao Huang, Yuanyuan Zhao, Miao Zhou, Yukang Guo, Haiyun Wang, Yuanyuan Chen**
*and Jian Peng**

Supporting Information

Ferrocene-Functionalized Black Phosphorus Nanoplatform Enables Targeted and Prolonged MRI Visualization of Atherosclerotic Plaques

Xiao Huang,[†] *Yuanyuan Zhao,*[†] *Miao Zhou,*[†] *Yukang Guo, Haiyun Wang, Yuanyuan Chen,*[*] *and Jian Peng*[*]

† These authors contributed equally to this work.

*Corresponding author: yychen@wust.edu.cn (Y.C.); jianpeng@whut.edu.cn (J. P.).

1. Materials and Methods

2. Supplementary Figures

1. Materials and Methods

1.1 Materials

Red phosphorus (RP), butylamine, tripropylamine, tin and tin iodide (SnI_4), aminoferrocene (AFc), hyaluronic acid (HA), Lipopolysaccharide (LPS), sulfo-Cyanine5.5 (Cy5.5) were purchased from Macklin Biochemical Co., Ltd (Shanghai). Dimethylformamide (DMF), dichloromethane (CH_2Cl_2) and other reaction reagents were purchased from Sinopharm Chemical Reagent Co. Ltd. (Shanghai, China). All chemicals were used without further purification. 3-(4,5-dimethylthiazol-2-yl)-2,5-diphenyltetrazolium bromide (MTT), Hoechst 33342 and Dimethyl sulfoxide (DMSO) were obtained by the biosharp Co., Ltd (Guangzhou, China).

1.2 Preparation of bulk black phosphorus (BP)

Bulk black phosphorus was prepared by a facile low-pressure transport method according to Xie's report.[s1] In a typical synthesis, 500 mg of RP, 20 mg of tin, and 10 mg of SnI_4 were sealed in an evacuated Pyrex tube. The tube was then heated at 1023 K for 5 h with a heating rate of about 1.35 K/min and then the temperature was decreased to 773 K with a cooling rate of 0.33 K/min, followed by a natural cooling process.

1.3 Preparation of 2D BP

The 2D BP was prepared by liquid exfoliation of corresponding bulk BP in DMF. In a typical preparation, 40 mg of bulk BP was dispersed in 40 mL of DMF that bubbled with argon to eliminate the dissolved oxygen molecules for avoiding oxidation, and the mixture solution was then tip-sonicated in ice water for 4 h and bath-sonication for another 4 h. Then, the resultant brown suspension was left to stand still for 12 h to allow any unstable aggregates to form and then the upper part of dispersion was collected for further modification.

1.4 Covalent functionalization of BP

Under nitrogen atmosphere, 40 mL of BP suspension (1 mg/mL) in DMF and 40 mL of CH_2Cl_2 were mixed at room temperature under stirring in a round-bottom flask. Then, 12 mg of AFc was added into the above mixture under vigorous stirring and purged with argon for another 30 min. The round-bottom flask with the mixture was heated by an oil bath under nitrogen atmosphere from room temperature to 100℃ for 30 min and kept at this temperature for 10 h with stirring. The resulting brown colloidal products were collected by centrifugation and washed three times with ethanol, then centrifuged at 10,000 rpm for 10 min. The supernatant was removed, and the precipitate was dried in vacuum. The black powder was re-dispersed in absolute ethanol before the characterization and stability test.

1.5 HA Modification

First, add 2 mg of FcP to 2 mL of PBS and sonicate for 15 minutes. Then 2 mg/mL HA was added to the mixture. After stirring overnight, centrifugation and washing were performed to the HA-FcP sample was obtained.

1.6 Characterization of samples

The ultraviolet-visible (UV–Vis) absorption spectra were recorded on a Shimadzu UV-1900i spectrometer equipped with 1-cm quartz cells. The spectra were recorded in the wavelength range of 200~1100 nm at a scan speed of 400 nm/min. Raman spectra (Xplora) were recorded with a He-Ne laser at the excitation wavelength of 532 nm. The FTIR spectra were obtained using a Fourier Transform Infrared Spectrometer (INVENIO R). X-ray diffraction (XRD, Rigaku/SmartLab SE) equipped with Cu Ka radiation (λ = 1.542 Å) over the 2θ range of 10–90° was used to characterize the structure of modified BP. The sample was prepared by depositing a film on the surface of a glass slide. The micrographs of samples were taken usingscanning electron microscope (SEM, ThermoFisher/Apreo S HiVac) with the accelerating voltage of 10 kV. X-ray photoelectron spectroscopy (XPS, AXIS SUPRA+) with an X−ray source of Mg Ka was used to study the composition of BP before and after modification. Xpspeak41 software was used to deconvolute the curves and fit the results. Thermal gravimetric analysis (TGA) was performed on an 449C/449F3 instrument under Ar atmosphere at a heating rate of 10 K/min. Dynamic light scattering and Zeta potential analysis were performed using a Malvern Nano−ZS. Atomic force microscope (AFM) images were acquired with contact mode in the air using Bruker/Dimension ICON. The EPR measurement was conducted on a Bruker EMXplus-6/1 EPR spectrometer. The freshly made sample tube was inserted into the measurement chamber and then put through the temperature of 100 K. The microwave frequency was set at 9.286 GHz with a power of 20 mW. For the measurement of the signals of FcP radicals, the center field was kept at 500G, with a sweep width of 300G. Each spectrum was integrated from 60 scans. The freshly made sample tube was inserted into the measurement chamber and then put through the temperature of 100 K.

1.7 Magnetic properties Measurement

The magnetic properties were measured with a Physical Property Measurement System (Lake Shore/8604) in the room temperature.

1.7 Cell Culture

RAW264.7 macrophages were purchasedfrom Procell. Cells were cultured in the standard cell culture medium at 37 °C under 5% CO_2 in Dulbecco's modified Eagle's medium (DMEM, high-glucose,GIBCO Invitrogen) supplemented with 1% penicillin/streptomycin and 10% fetal bovine serum (FBS). Macrophages were activated by 1 μg/mL LPS incubation for 24 h.

1.8 Cytotoxicity Evaluation

Macrophages were seeded in 96-well plates and incubated with different concentrations of HA-FcP for 24h. In briefly, 10 μL of 5 mg/mL MTT solution was added to each well in a 96-well cell culture plate and incubated in an incubator for 4 h. After blue crystals appeared in the wells, the liquid was aspirated, and 150 μL of DMSO was added to each well, and the precipitate was dissolved by slow oscillation for 10 min, and the absorbance at 490 nm of each well was measured by an enzyme marker.

1.9 Cellular Uptake Analysis

Activated macrophages was seeded into a 24-well plate at a density of 5000 cells/well and incubated with fluorescent Cy5.5-labeled HA-FcP (HA-FcP-Cy5.5) or fluorescent Cy5.5-labeled FcP (FcP-Cy5.5) at 37°C for 6 h. The cells were fixed with 4% paraformaldehyde solution and nuclei stained with Hoechst 33342 for 10 min before confocal laser scanning microscope (CLSM) observation.

1.10 Animal Model

All animal experiments were approved by the Wuhan University of Science and Technology University (ethicalapproval number 2023038). ApoE$^{-/-}$ mice (6−8 weeks old, male) were purchased from Huachuang Sino (JiangShu, China). Mice were housed in ventilated cages under specific pathogen-free conditions with a 12 h light/dark cycle and fed with a high-fat diet. To induce the carotid atherosclerotic lesions, left external and internal carotid arterial branches were ligated on day 14, whereas the right carotid artery received sham surgery.

1.11 In Vivo Localization of HA-FcP

Fourteen days after partial carotid ligation surgery, mice were randomized to two groups and intravenously injected with Cy5.5-FcP or Cy5.5-HA-FcP at the concentration of 10 mg/kg. At different time points (3, 6, 9, 12 and 24 h), Using a PerkinElmer in vivo imaging system (IVIS) Spectrum for in vivo imaging of mice the left and right carotid arteries were collected and imaged with a PerkinElmer IVIS Spectrum. A fluorescence signal was used to analyze the accumulation of HA-FcP in lesions quantitatively.

1.12 In Vivo MRI Screening of Plaques in ApoE$^{-/-}$ Mice

Images were obtained using a 9.4 T (Bruker 94/30 USR) small animal magnet with a birdcage coil having a diameter of 30 cm. Mice were anesthetized with 1.5% isoflurane air mixture at 35–37°C with warm air flowing through the bore, and respiration was monitored (MP150, Biopac, Goleta, CA). Intravenous injection of 10 mg/kg BP and HA-FcP via the tail vein for in vivo MRI. The imaging was performed prior to the injection and at 3, 6, 24, 48, 72 and 120 h post-injection.

1.13 Histopathological Examination

After different treatments, mice were sacrificed on day 42. The left carotid arteries were harvested and fixed in 4% paraformaldehyde. After being embedded in paraffin, the left carotid arteries were sectioned into slices for hematoxylin and eosin (H&E) staining and stained with Oil red O. The specimens were imaged by an optical microscope and analyzed with ImageJ.

1.14 Hemolysis Assay

HA-FcP PBS solutions were mixed with mouse red blood cells at different concentrations (0, 50, 100 and 200 μg/mL). For comparison, deionized water was used as a positive control. After incubation at 37°C for 1 h, the mixtures were centrifugated (1000 g, 15 min). Then the absorbance of the supernatant was measured by a microplate reader at the wavelength of 576 nm.

1.15 Biodistribution analysis

2 groups of 6-week-old female C57BL/6 mice (n = 3 in each group) were i.v. injected with saline and HA-FcP, respectively. The body weight was monitored for 15 days after postinjection. Finally, major organs in each group were harvested, fixed in 4% paraformaldehyde, embedded in paraffin, and then sectioned into slices for H&E staining. In addition, the blood serum was collected for blood chemistry analysis, including the liver function biomarkers (ALT and AST) and kidney function biomarkers (BUN and UA) analysis, as well as inflammatory cytokines (TNF-α, IL-6, and IL-1β) analysis.

1.16 Statistical Analysis

All data were expressed as "mean ± standard deviation (SD)". Data analysis by Student's t-test: $*p < 0.05$, $**p < 0.01$, $***p < 0.001$, $****p < 0.0001$, ns: not significant.

2. Supplementary Figures and Tables

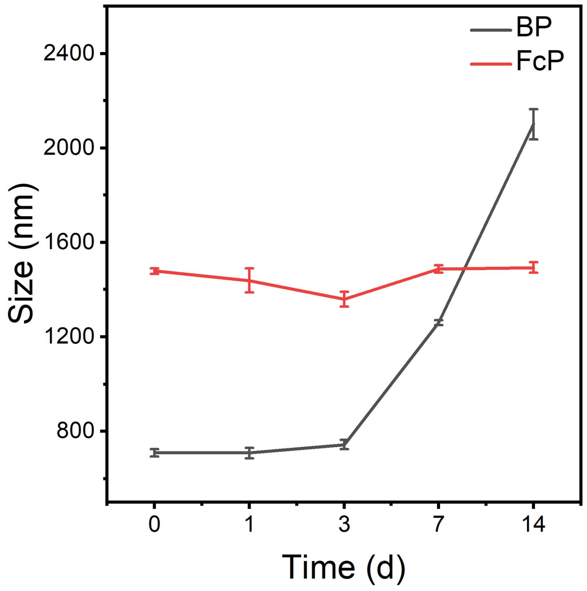

Figure S1. Changes in particle size of BP and FcP over 14 d.

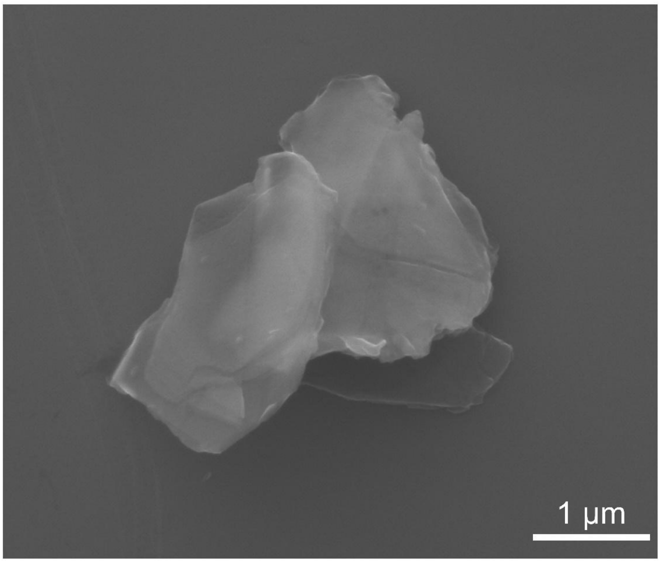

Figure S2. SEM image of FcP.

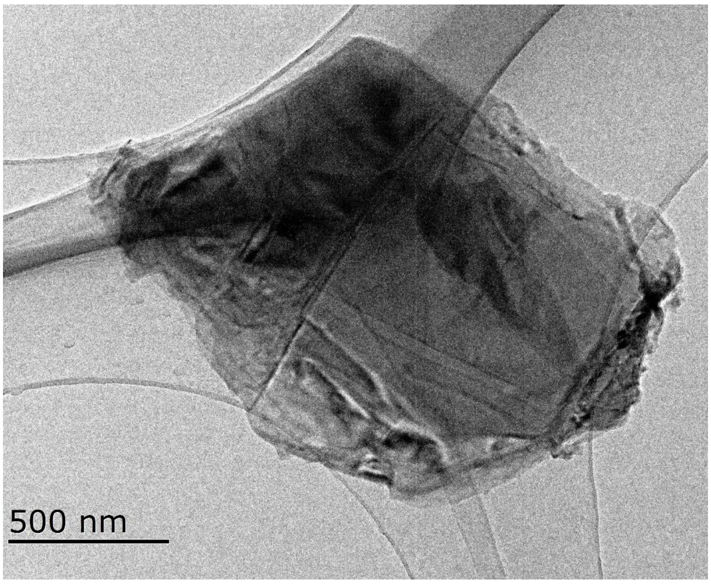

Figure S3. TEM image of FcP.

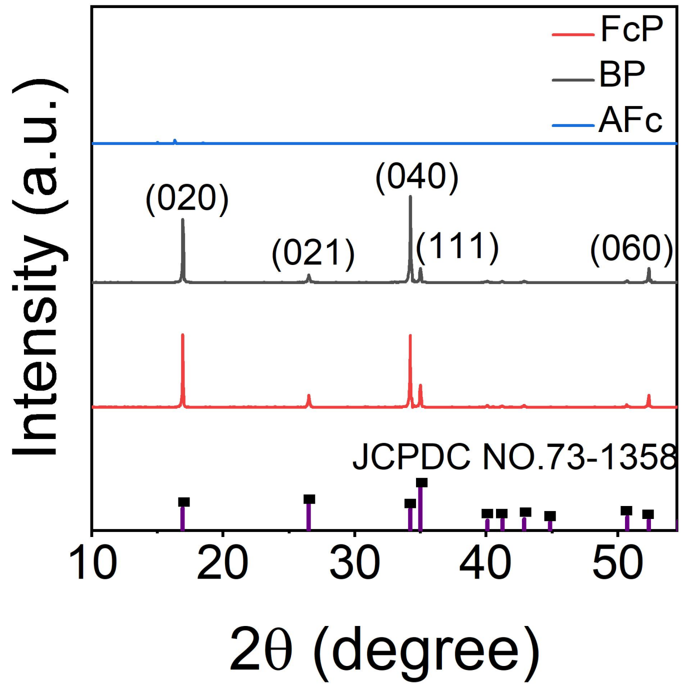

Figure S4. XRD spectra of FcP, BP and AFc.

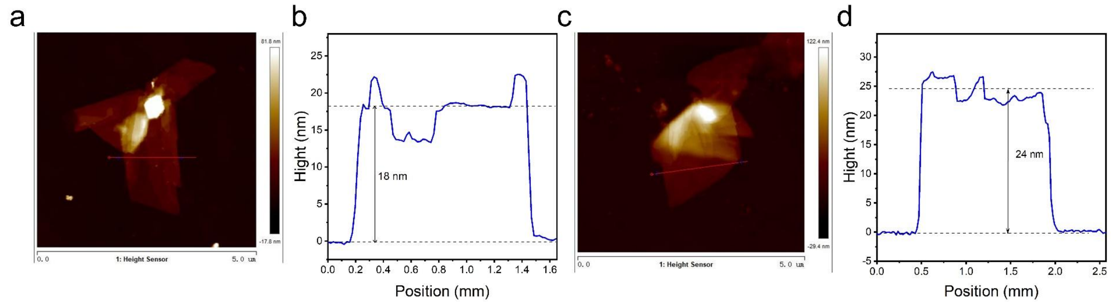

Figure S5. Characterization of FcP. AFM image (a, c) and corresponding height profile (b, d) along the red line in (a, c).

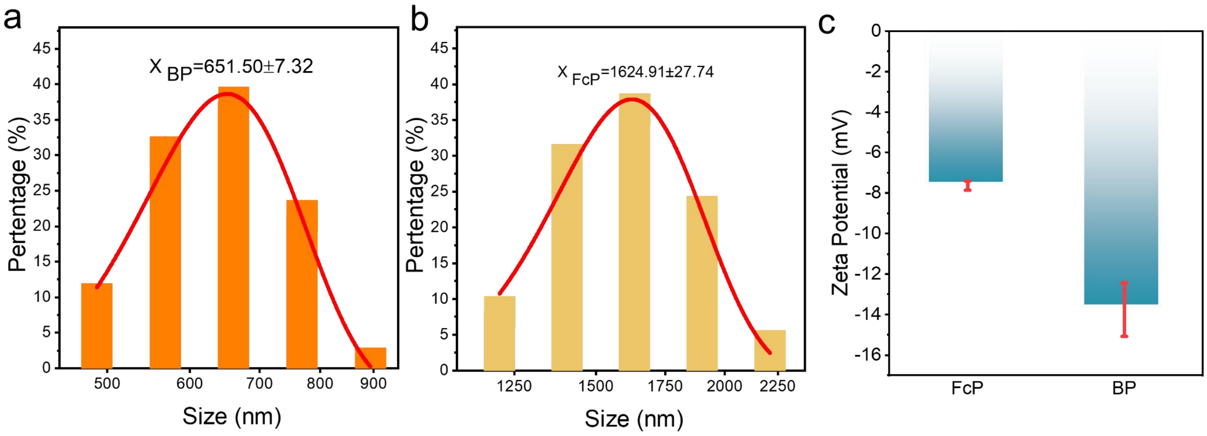

Figure S6. Characterization of FcP. (a) DLS of BP (b) DLS of FcP (c) Zeta potential of BP, FcP in aqueous solutions.

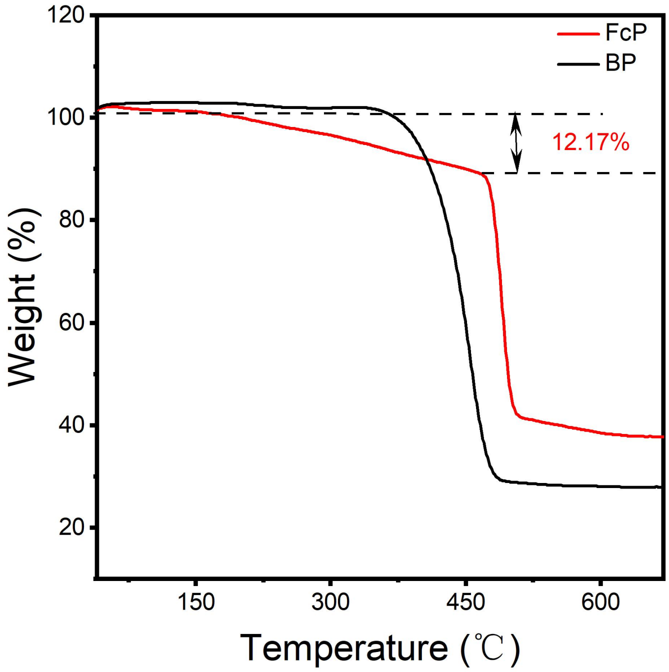

Figure S7. TGA of FcP and BP.

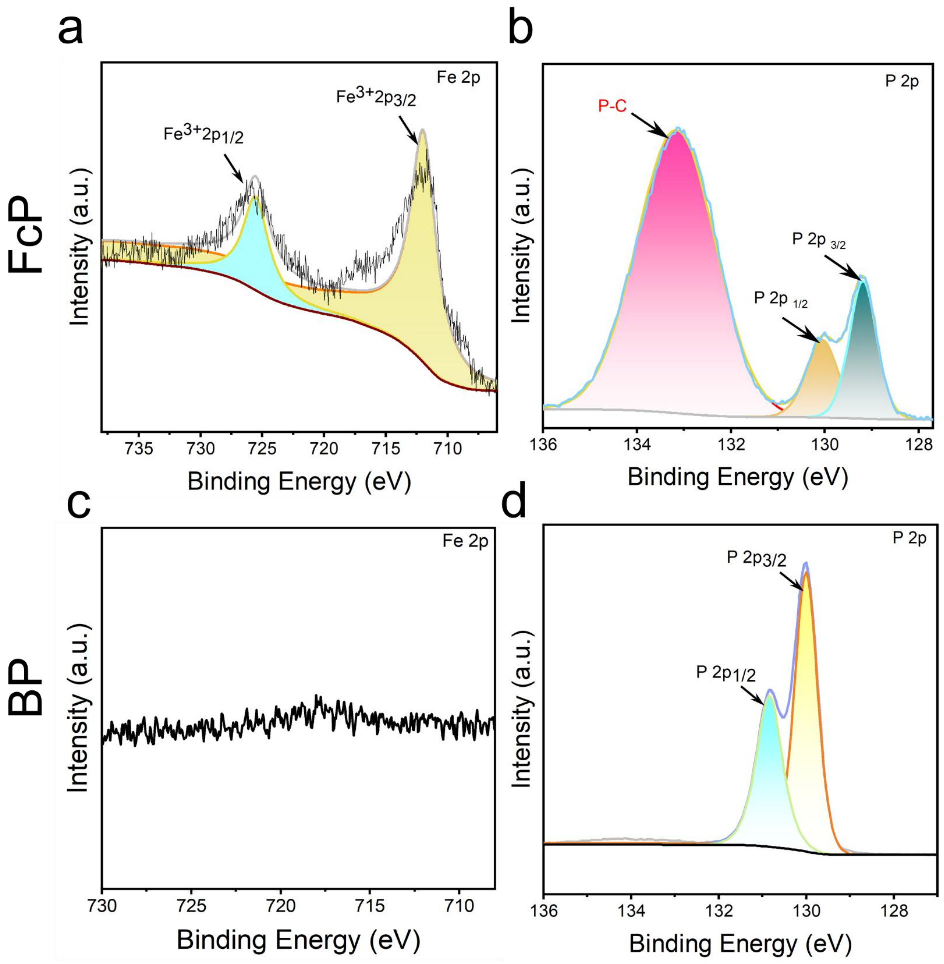

Figure S8. Covalent functionalization of BP by AFc. XPS high-resolution Fe 2p, P 2p spectre of FcP (a-b) and XPS high-resolution Fe 2p and P 2p spectra of BP (c-d).

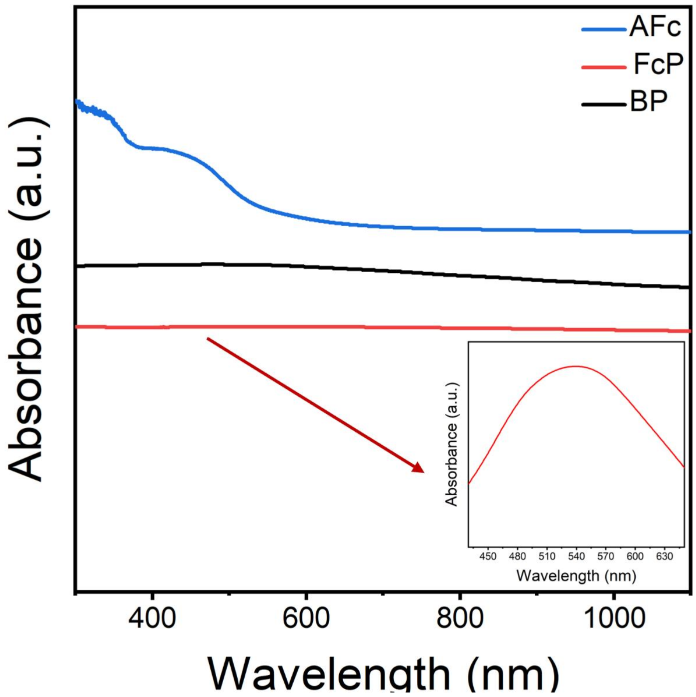

Figure S9. UV absorption spectrum of FcP, BP and AFc. The inset shows the UV absorption of FcP at wavelengths of 450-630 nm.

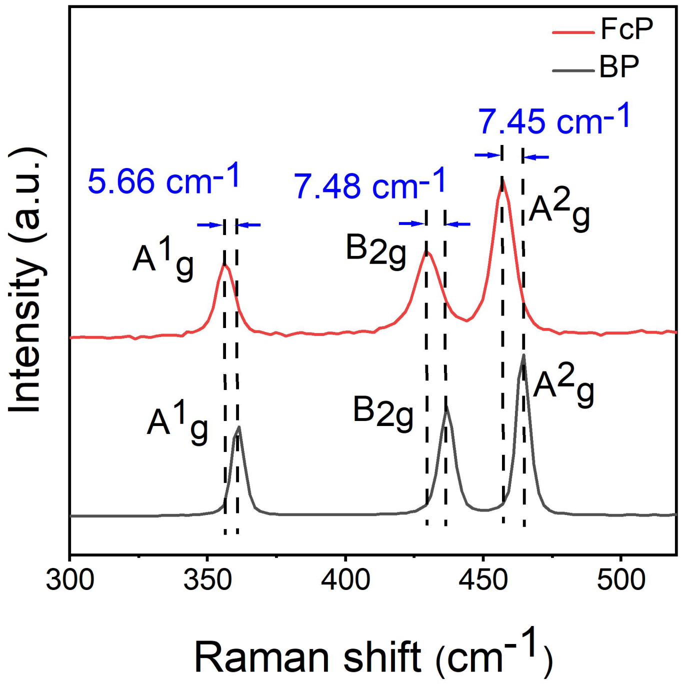

Figure S10. Raman spectra of FcP, BP and AFc.

Table S1 Mössbauer Parameters Obtained from the Fit of the Spectra Shown in Figure 1f.

Sample name	IS (mm/s)	QS (mm/s)	Γ/2 (mm/s)
FcP	0.29	0.5	0.28

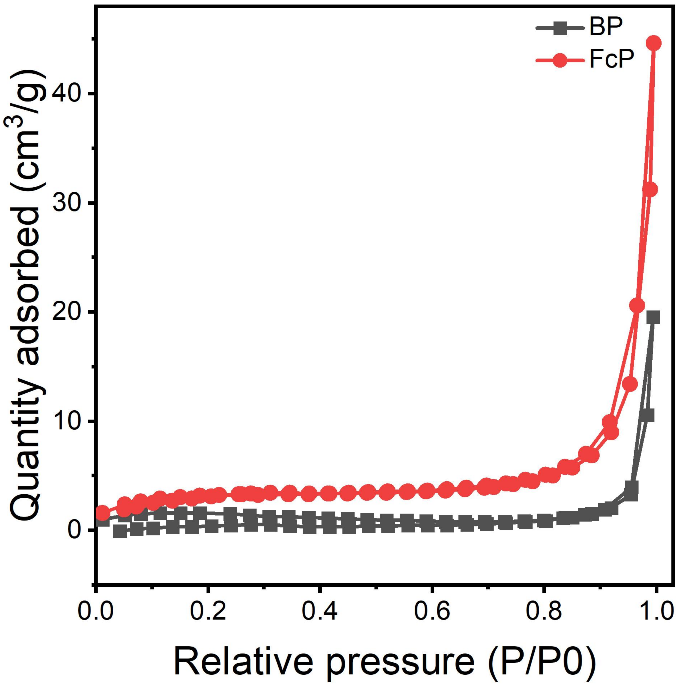

Figure S11. N_2 adsorption isotherm of the FcP and BP at 77 K.

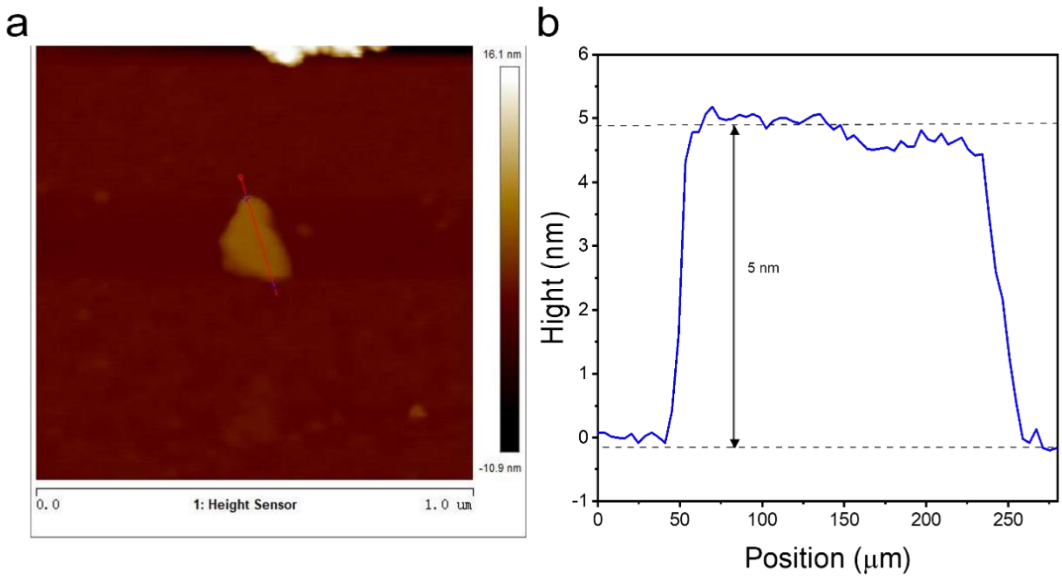

Figure S12. Characterization of BP. (a)AFM image of BP; (b) corresponding height profile (a) along the red line.

Assuming an ideal model for a single layer BP with a 2000 nm length × 1000 nm width rectangle, and the number of P atoms per nm^2 is 27.49 atoms.[s2] As measured by the AFM (Figure S12), BP has a thickness of 10 layers. The phosphorous atoms of upper and lower faces will react with AFc and internal atoms will not react with AFc. It can be calculated that the number of surface phosphorus atoms in a 2000 nm × 1000 nm rectangular BP of 40 layers = single-layer BP area × 2 layers × the number of P atoms per nm^2= 2000 nm×1000 nm ×2 × 27.49/nm^2= 1.1×10^8. The total amount of reacted phosphorous substance = the number of surface P atoms / avogadro's constant =1.1×10^8/NA≈1.1 $\times10^8$/NA (NA: Avogadro's constant). AFc radical consists of basic units of C_6H_7FeN. The diameter of the basic unit is 4.9 Å. If the surface of BP nanosheet is completely covered by a unit layer of AFc radicals, then the number of basic units of AFc required = BP surface area / basic unit of AFc = 2000 nm ×1000 nm ×2 / (3.14×0.245 nm ×0.245 nm) ≈ 2.12×10^7. The amount of AFc substance = the number of basic units/NA≈2.12×10^7/NA. Therefore, when the surface of the 10-layer BP is covered by one unit layer of AFc, the ratio of the amount of P/AFc required = the amount of surface phosphorus substance/the amount of AFc substance in each basic unit =1.1×10^8/NA/2.12×10^7/NA≈ 4:1.

To determine the grafting density of AFc molecules on the BP surface in the experiment, FcP and BP samples were each characterized by surface area. The method of calculation for AFc molecular content is as follows: The specific surface area of FcP obtained by the BET test is 12.0516 m^2/g (Figure S11), combined with the mass fraction 2.12% of nitrogen in FcP measure by element analysis, which is converted to a molar concentration of 0.0015 mol nitrogen atoms/g. The grafting density after the AFc reaction is calculated to be 6.276 AFc/nm^2, with the number of P atoms per nm^2 being 27.49/nm^2. Combined, the molar ratio of P atoms to AFc molecules is measured in the experiment to be 4.38:1. With an ideal full coverage model, the ratio is 4:1. Therefore, the AFc molecules fully cover the BP surface (Figure S13). That is, one N-C binds one phosphorus atom to form a P-C-N bond.

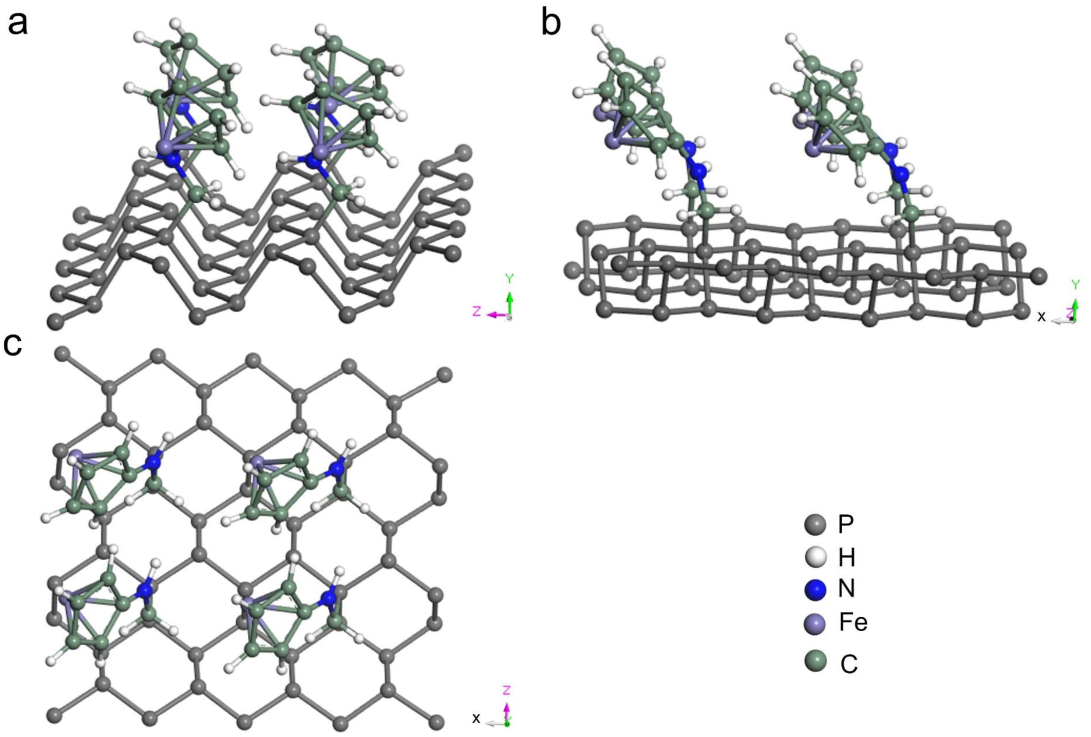

Figure S13. The proposed atom model of FcP from the side view(a-b). The proposed atom model of FcP from the top view(c).

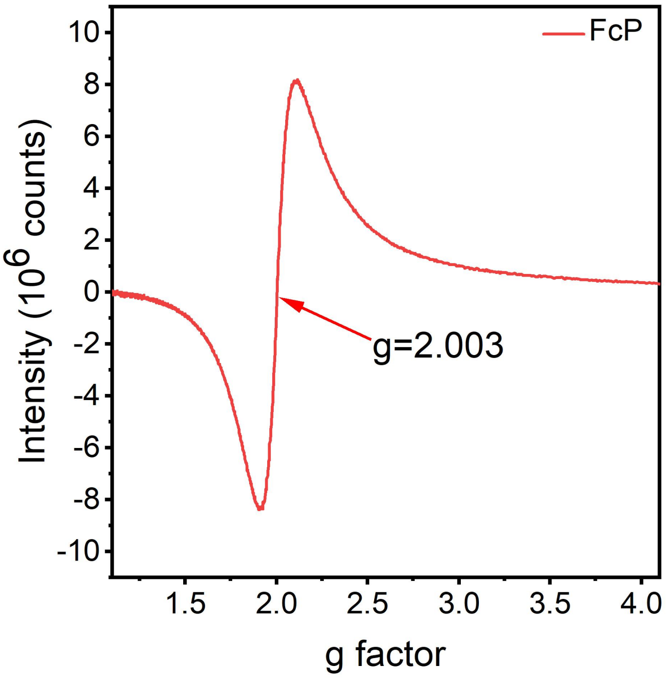

Figure S14. EPR spectra of FcP.

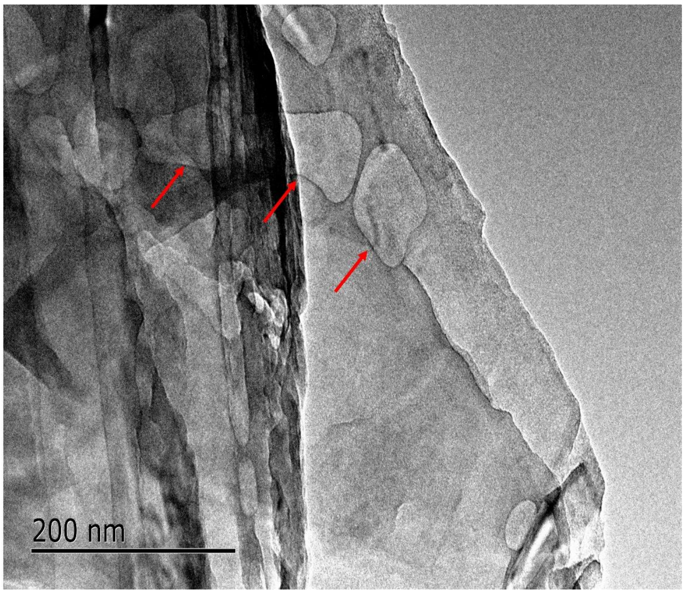

Figure S15. TEM image of HA-FcP, the part indicated by the red arrow is HA.

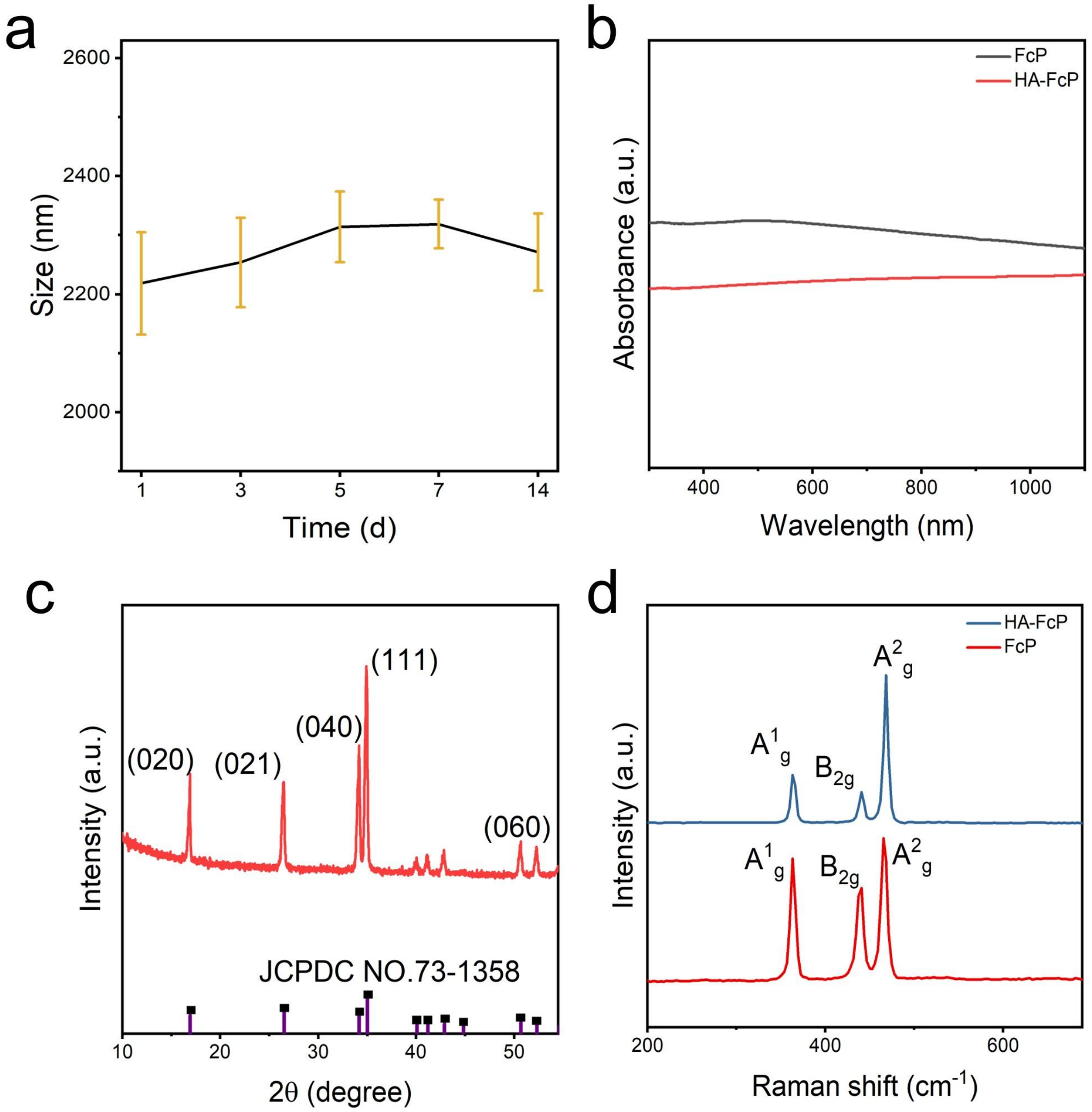

Figure S16. Characterizations of HA-FcP. (a) Changes in particle size of HA-FcP over 14 d. (b) UV absorption spectrum of HA-FcP. (c) XRD spectra of HA-FcP. (d) Raman spectra of HA-FcP.

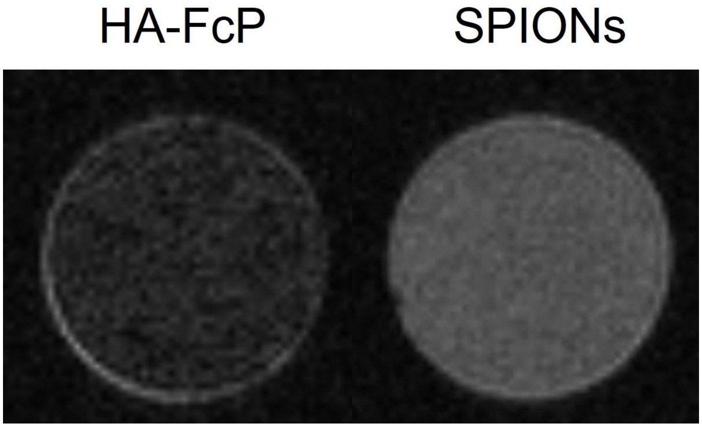

Figure S17. T_2-weighted images of HA-FcP and SPIONs (Fe concentration is 0.32 mM).

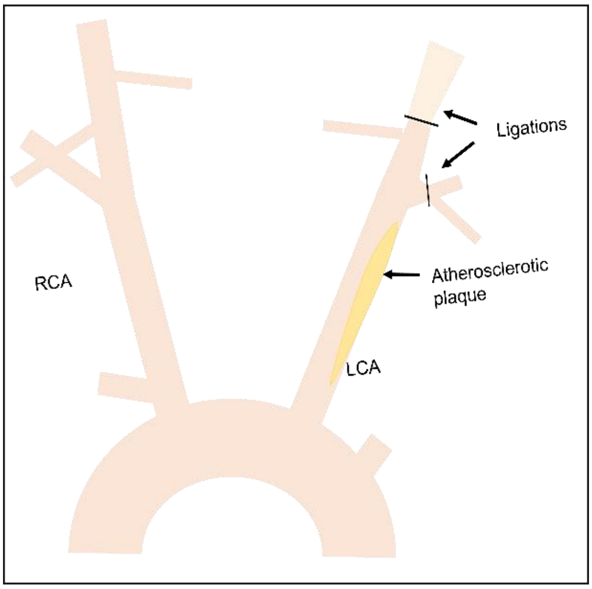

Figure S18. Schematic illustration of partial left carotid artery ligation.

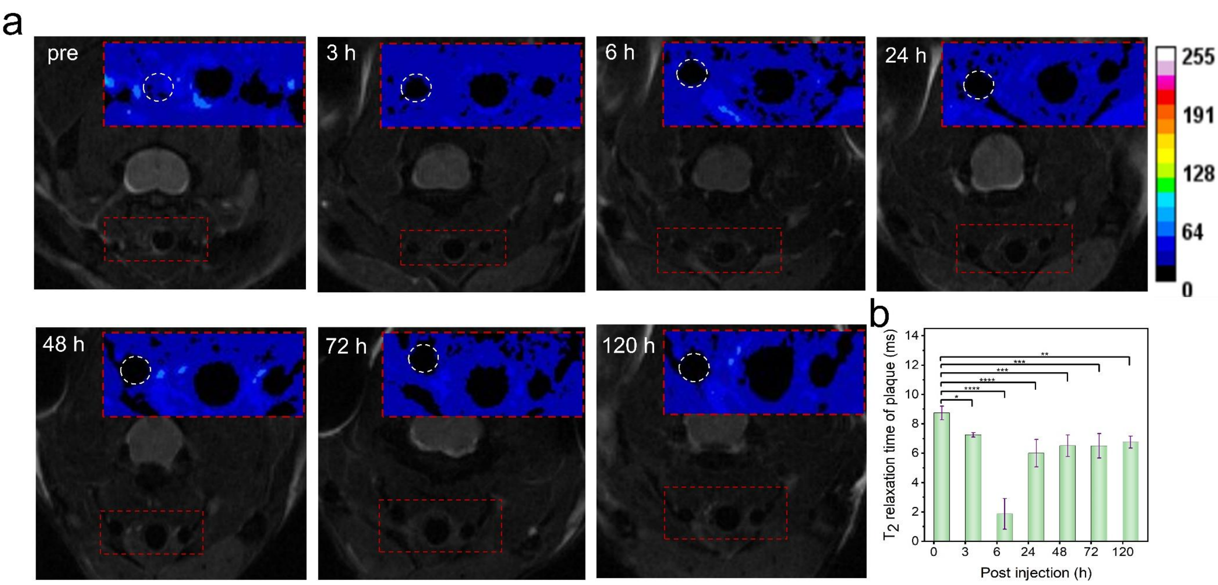

Figure S19. Imaging of HA-FcP in moderate aorta AS models. (a) MRI of atherosclerotic aorta at different time points of HA-FcP injection (pre-injection; 3, 6, 24, 48, 72 and 120 h postinjection). The vessel wall is indicated with a white circle, and plaque is indicated with a white circle. (b) Quantification of T2 relaxation of plaque. Data are presented as mean ± SD (n=5). **p* < 0.05 was considered significant, and ***p* < 0.01, ****p* < 0.01 were considered highly significant.

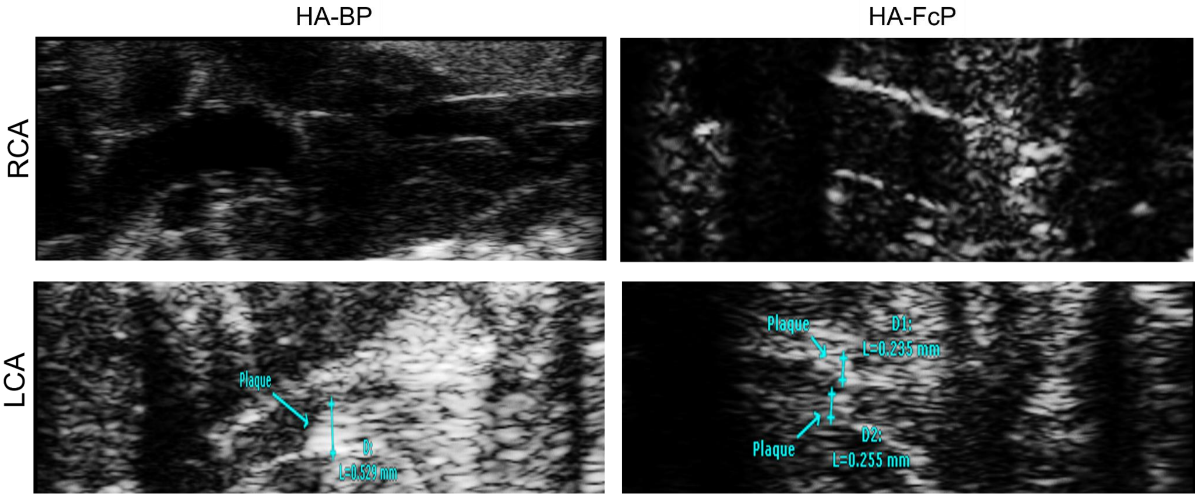

Figure S20. ultrasonography image of the plaque.

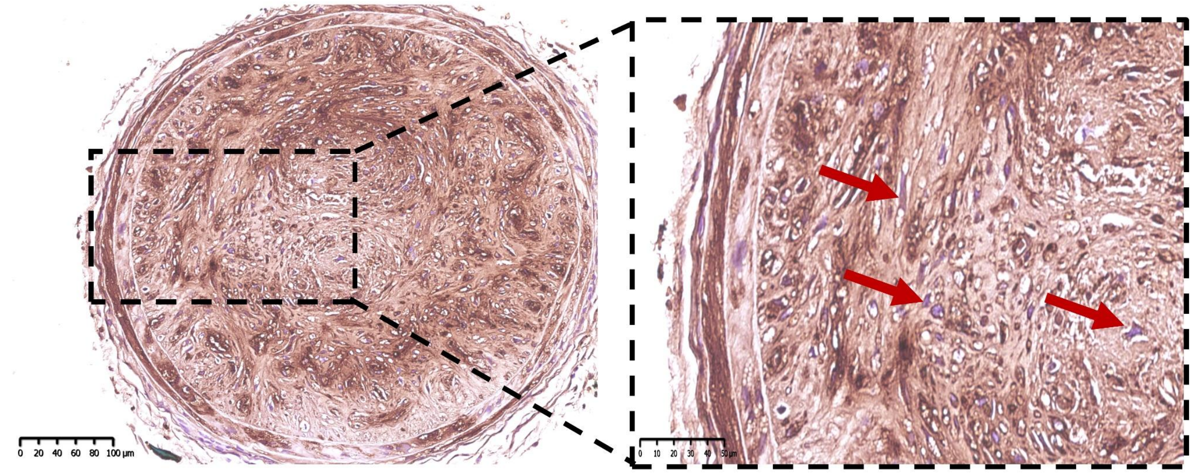

Figure S21. Prussian blue staining of plaques after MRI detection, the blue areas indicated by the red arrows represent iron ions deposited within the plaque regions.

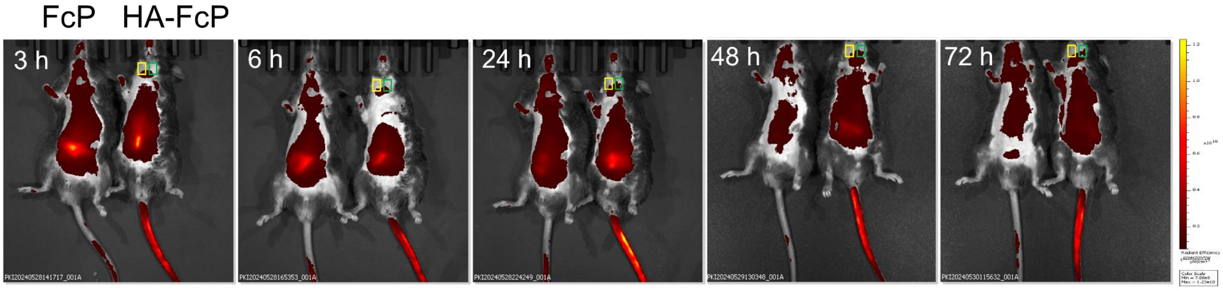

Figure S22. In vivo fluorescence imaging at different time intervals after FcP and HA-FcP injection respectively (the green box:LCA, the yellow box: RCA).

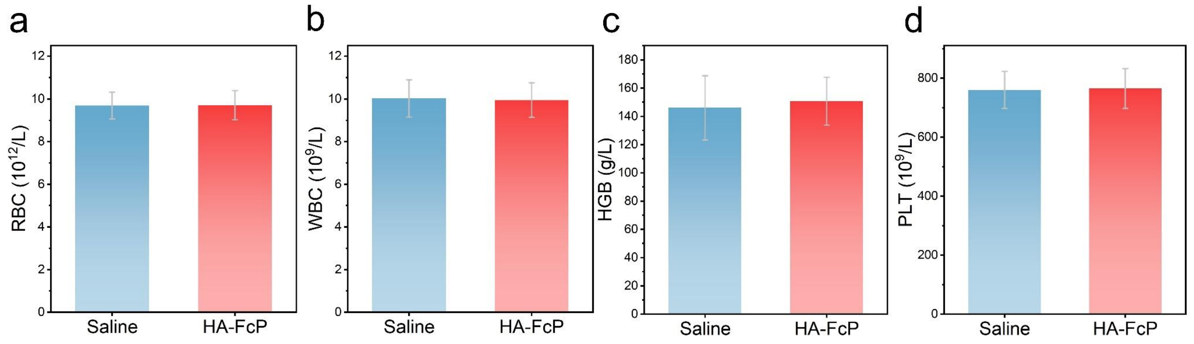

Figure S23. Typical hematological parameters. (a) RBC, red blood cell. (b) WBC, white blood cell. (c) HGB, hemoglobin. (d) PLT, platelet. The data are shown as means ± SD (n = 3).

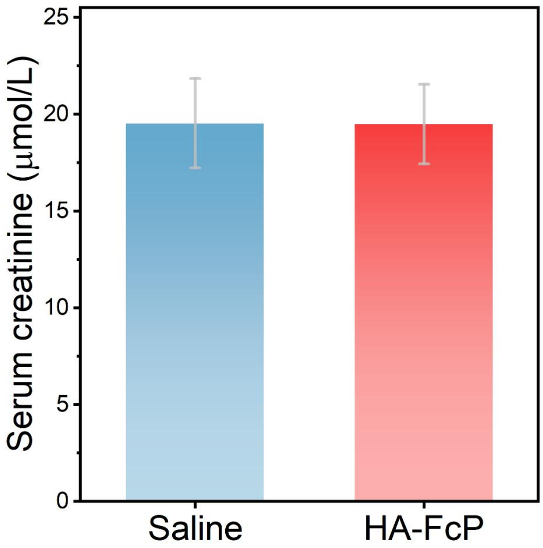

Figure S24. Renal function indicator (serum creatinine) in the serum of C57BL/6 mice.

References

[s1] H. Wang, X. Yang, W. Shao, S. Chen, J. Xie, X. Zhang, J. Wang, Y. Xie, *J. Am. Chem. Soc.* **2015**, *137*, 11376.

[s2] X. Liu, P. Fan, L. Xiao, J. Weng, Q. Xu, J. Xu, *J. Energy Chem.* **2021**, *53*, 185.
